# Nutritional pressure from serum amplifies dysbiotic features in an oral microbiome synthetic community

**DOI:** 10.1093/ismejo/wrag070

**Published:** 2026-03-27

**Authors:** Lu Li, Matthew Smardz, Dam Soh, Philip D Marsh, Anilei Hoare, Patricia I Diaz

**Affiliations:** UB Microbiome Center, State University of New York at Buffalo, Buffalo, NY 14214, United States; Department of Oral Biology, School of Dental Medicine, State University of New York at Buffalo, Buffalo, NY 14214, United States; UB Microbiome Center, State University of New York at Buffalo, Buffalo, NY 14214, United States; Department of Oral Biology, School of Dental Medicine, State University of New York at Buffalo, Buffalo, NY 14214, United States; UB Microbiome Center, State University of New York at Buffalo, Buffalo, NY 14214, United States; Department of Oral Biology, School of Dental Medicine, State University of New York at Buffalo, Buffalo, NY 14214, United States; School of Dentistry, University of Leeds, Leeds, LS2 9JT, United Kingdom; Laboratory of Oral Microbiota Ecology, Faculty of Dentistry, Universidad Andres Bello, Santiago, Santiago 8320000, Chile; UB Microbiome Center, State University of New York at Buffalo, Buffalo, NY 14214, United States; Department of Oral Biology, School of Dental Medicine, State University of New York at Buffalo, Buffalo, NY 14214, United States

**Keywords:** synthetic community, oral microbiome, subgingival, periodontitis, chemostat, serum, gingival crevicular fluid, nutritional substrate

## Abstract

Despite rapid advances in characterizing the human microbiome, the ecological pressures shaping its transitions from healthy to diseased states remain poorly resolved. This is particularly true for periodontitis, a slow-progressing chronic inflammatory disease associated with well-defined shifts in the subgingival microbiome. Here, we report the development of a complex synthetic community model of the subgingival microbiome, designed for systematic interrogation of ecological factors that drive community restructuring. The model includes 22 prevalent and abundant subgingival species maintained in mucin-rich medium under microaerophilic, continuous culture conditions, in a chemostat. Using this system, we interrogated the impact of serum, as a surrogate for the inflammatory exudate produced by the host in response to biofilm accumulation, on community structure and function. Through integrated 16S rRNA gene sequencing, metatranscriptomics, and metabolomics, we found that serum was not required for a community with a periodontitis-like configuration to establish, but its presence intensified features of dysbiosis. Serum increased total biomass, promoted polymicrobial aggregate formation, promoted nitrogen and protein metabolism thereby modifying the environmental pH towards alkalinity, and introduced nitrosative stress. Serum also modified the community metatranscriptome in ways that paralleled microbiome activities in human periodontitis. Serum, however, decreased community diversity by disproportionally conferring a competitive advantage to the pathogen *Porphyromonas gingivalis*. This synthetic community model has revealed serum as a key nutritional pressure that modulates subgingival microbiome ecology and may perpetuate dysbiosis.

## Introduction

Recent omics-based studies of human associated microbiomes have advanced, in an unprecedented manner, our understanding of the disruptions in host-microbiome homeostasis that accompany a wide-spectrum of diseases [1–4]. In the oral cavity, the chronic inflammatory disease periodontitis is intricately linked to profound compositional and functional shifts in the community of microorganisms residing at the gingival crevice (the subgingival microbiome). Periodontitis-associated microbial communities are generally more diverse, enriched with numerous taxa—predominantly Gram-negative anaerobes—and exhibit higher microbial biomass than health-associated communities [5–7]. The relationship between microbial dysbiosis and destructive inflammation of the periodontal tissues is thought to be part of a self-perpetuating cycle in which enrichment of pathobionts at the gingiva triggers a dysregulated host response causing tissue inflammation and damage, which in turn alters the subgingival environment driving further pathobiont enrichment [8, 9]. Understanding of the ecological drivers of subgingival community dysbiosis is, however, still incomplete, which has resulted in lack of successful therapies to restore communities to a health-compatible configuration [10].

The evolution of microbial communities during periodontitis is driven by interactions among its members and of microorganisms with the host environment, ultimately generating a niche conducive to pathobiont expansion. Investigating these processes directly in humans remains difficult because periodontitis progresses slowly, and even with extensive longitudinal follow up of subjects and analyses of time-series data [11], it is nearly impossible to disentangle the contributions of individual microorganisms or environmental factors to community dynamics. Previous studies have demonstrated that inoculating natural oral or fecal microbiome communities into in vitro static systems enables inquiries regarding the role of the nutritional environment on community composition [12–15]. Although these models are valuable for probing factors that influence dysbiosis, natural communities vary substantially across hosts and lack the experimental manipulability required to mechanistically evaluate the roles of specific members, or their genes, as determinants of community outcomes. Conversely, synthetic communities are reproducible experimental systems in which community composition can be systematically manipulated. In previous work, we employed synthetic communities of up to 10 oral species to assess how the presence or absence of specific taxa influenced community composition [16, 17]. These studies integrated defined communities with a chemostat continuous culture system, in which nutrient influx rates can be modulated to support growth of slow-dividing species, thus overcoming a known limitation of static batch models. This platform also enables repeated sampling for longitudinal monitoring and permits precise control of environmental parameters, allowing systematic evaluation of the influence of individual factors on community properties. Microbiome shifts associated with periodontitis development are well defined, at least from a taxonomic perspective [4–7], with recent insight into community transcriptional activities as periodontitis progresses [11, 18]. Consequently, developing a complex synthetic community that models the subgingival microbiome represents a logical next step to rigorously dissect the ecological drivers of dysbiosis.

One of the main environmental influences thought to drive subgingival microbiome dysbiosis is gingival crevicular fluid (GCF), an exudate produced in response to biofilm accumulation at the gingival crevice. GCF bathes subgingival communities and contains serum-derived glycoproteins as well as tissue-produced inflammatory and breakdown products [19–21]. Biofilm-driven inflammation of the periodontal tissues increases the flow rate of GCF [22, 23]. This inflammatory exudate is thought to serve as a nutritional selective force that promotes dysbiotic shifts, as suggested by previous studies in which natural subgingival communities were cultivated in closed static systems, where the presence of serum—used as a surrogate for GCF—enriched certain bacteria typical of inflamed periodontal pockets [12–14, 24]. However, the manner in which serum modifies community metabolism and function, and whether this nutritional resource is the main ecological driver promoting the assembly of periodontitis-associated communities, are concepts still incompletely understood. Here, we describe the development of a complex synthetic subgingival community comprised of 22 species maintained in a chemostat under continuous culture and demonstrate that, when coupled with “omics” analytical techniques, this model provides a powerful platform to dissect community interactions with specific environmental factors. We used this model to investigate the nutritional impact of serum on microbial community composition and function. Our work shows that serum, irrespective of concentration, promoted communities with greater biomass and provided a clear competitive advantage to the pathogen *Porphyromonas gingivalis*. Elevated serum levels also enhanced planktonic aggregate formation and switched community metabolism promoting ammonia production and a raise in the environmental pH, partly due to the activities of *P. gingivalis*. Higher serum also resulted in community-wide changes in expression of genes involved in protein and DNA repair, suggestive of a response to nitrosative stress, and modified community transcriptional activities in a manner that partly paralleled the microbiome functional activities in human periodontitis. Serum, however, was not necessary for the establishment of a community with a periodontitis-like configuration. Collectively, these studies suggest that serum functions as a nutritional driver that exacerbates subgingival microbiome dysbiosis.

## Materials and methods

### Preparation of inoculum for synthetic community

**Figure 1:**
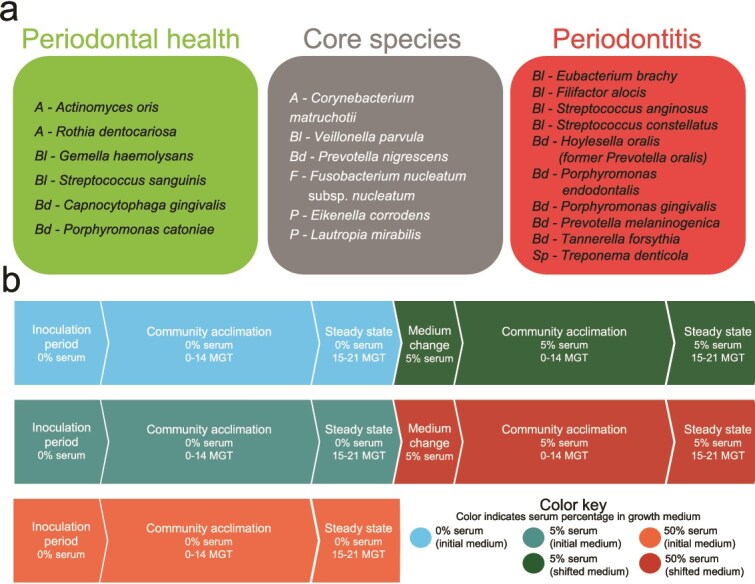
Overview of the synthetic community and experimental design for chemostat experiments. (a) Inocula for the synthetic community included 22 highly prevalent and abundant subgingival species associated with periodontal health, periodontitis, or non-associated (core species). Species are shown with a prefix denoting their phylum: *A* = *Actinomycetota*, *Bl* = *Bacillota*, *Bd* = *Bacteroidota*, *F* = *Fusobacteriota*, *P* = *Pseudomonadota*, *Sp* = *Spirochaetota*. (b) Flowchart for the three different experimental designs used to test the role of serum on community properties. Medium flow was started after the inoculation period. MGT indicates the number of generations monitored for each specific period.

Twenty-two bacterial species commonly found in subgingival biofilms were selected based on their prevalence and abundance in periodontally healthy or periodontitis-affected individuals, as reported in our previous studies (Fig. 1a and Supplementary Table S1) [4, 5]. The strains *Actinomyces oris* T14V, *Rothia dentocariosa* ATCC 17931, *Gemella haemolysans* ATCC 10379, *Streptococcus sanguinis* SK36, *Capnocytophaga gingivalis* ATCC 33624, and *Porphyromonas catoniae* F0037 were selected as health-associated species. *Corynebacterium matruchotii* ATCC 33806, *Veillonella parvula* PK1910, *Prevotella nigrescens* ATCC 33563, *Fusobacterium nucleatum* subsp. *nucleatum* ATCC 25586, *Eikenella corrodens* ATCC 23834, and *Lautropia mirabilis* ATCC 51599 were selected to represent core species, which are prevalent and abundant subgingival taxa irrespective of periodontal health status. Periodontitis-associated species included *Eubacterium brachy* ATCC 33089, *Filifactor alocis* ATCC 35896, *Streptococcus anginosus* ATCC 33397, *Streptococcus constellatus* ATCC 27823, *Hoylesella oralis* ATCC 33269 (formerly *Prevotella oralis*), *Porphyromonas gingivalis* W83, *Porphyromonas endodontalis* ATCC 35406, *Prevotella melaninogenica* ATCC 25845, *Tannerella forsythia* ATCC 43037, and *Treponema denticola* ATCC 35405 (Fig. 1a). Each strain was maintained at 37°C in the appropriate medium and atmospheric conditions (Supplementary Table S2).

For chemostat community assembly, standardized frozen inocula were prepared from starter cultures grown in the specific medium for each microorganism. Cultures were grown until mid exponential phase, and then normalized to OD_600_ = 0.7, equivalent to ~10^9^ cells ml^−1^. Aliquots of 20 ml were collected and centrifuged at 1857 × g for 15 min. The resulting pellets were resuspended in 500 μL of growth medium supplemented with 10% glycerol, transferred into cryovials, frozen at −80°C for 24 h, and subsequently stored in liquid nitrogen until further use.

### Continuous culture conditions

Continuous culture experiments were conducted in a Bioflow/CelliGen 115 Bioreactor (New Brunswick Scientific,) using previously prepared frozen inocula of each species, which were combined and introduced into 400 ml of mucin-serum medium [25]. The medium contained 2.5 mg ml^−1^ hog gastric mucin (Sigma-Aldrich), 2.5 mg ml^−1^ KCl, 2 mg ml^−1^ proteose peptone, 1 mg ml^−1^ yeast extract, 1 mg ml^−1^ trypticase peptone, 0.1 mg ml^−1^ cysteine·HCl, and was supplemented after autoclaving with 10 μg ml^−1^ N-acetylmuramic acid (NAM), 5 μg ml^−1^ hemin and heat-inactivated horse serum (Gibco, Thermo Fisher Scientific) to final concentrations of 0%, 5%, or 50% (v/v). Antifoam 204 (Sigma-Aldrich) was added to a final concentration of 0.005% prior to autoclaving. Inoculation was performed as previously described [17], including an initial inoculation followed by 24 h of batch growth and 24 h of continuous culture, and a second inoculation followed by 24 h of batch growth after which continuous culture was resumed (this was considered day 0). A microaerophilic atmosphere was introduced by continuously sparging the culture with a gas mix consisting of 2% O_2_, 5% CO_2_ and 93% N_2_. Temperature and pH were automatically controlled at 37°C and 7.15 ± 0.15, respectively.

Cultures were monitored daily by measuring OD, dry weight, and examined using phase contrast microscopy. In addition, redox potential (*E*_h_), dissolved oxygen, and the volume of acid and base added were recorded daily. Cultures were considered to have reached steady-state after 15 mean generation times (MGT), with evidence of sustained stability based on dry weights and *E*_h_ measurements.

Two pilot experiments were performed to determine the optimal conditions for the growth of species with varying growth rates (Supplementary Fig. S1). Five-species communities, consisting of *P. gingivalis*, *F. nucleatum* subsp. *nucleatum*, *V. parvula*, *A. oris*, and *S. sanguinis* were established in continuous culture at two different dilution rates. The first protocol was based on dilution rates previously used to grow oral communities [16] with a doubling time (Td) of 6.7 h, dilution rate (D) of 0.103 h^−1^, and flow rate (F) of 51.56 ml h^−1^. The second protocol was based on the experimental growth rate of *P. gingivalis* in mucin-serum (Td = 15 h), using a D = 0.0462 h^−1^, and a F = 23.21 ml h^−1^. Colony-forming unit (CFU) counts were obtained for *A. oris*, *S. sanguinis*, *F. nucleatum*, and *V. parvula*, and *P. gingivalis* abundance was assessed by quantitative real-time PCR (qPCR) targeting the 16S rRNA gene [26]. *P. gingivalis* exhibited low biomass under the 6.7 h Td protocol compared with the other four species (Supplementary Fig. S1). In contrast, under the 15 h Td condition, the biomass of *P. gingivalis* increased by ~2.5 log whereas other species maintained high cell densities. Based on these results, and considering the synthetic community included other slow-growing species, the Td = 15 h protocol was selected for subsequent experiments.

Three types of experiments were conducted to evaluate the effect of serum on the 22-species community (Fig. 1b). First, the community was established without serum and subsequently shifted to 5% serum after reaching theoretical steady-state (15–21 MGT), after which the culture was monitored until a second steady-state was achieved. Alternatively, the community was assembled in the presence of 5% serum and shifted to 50% serum after steady-state. In the third type of experiment, the community was established and maintained at 50% serum. In all cases, the following samples were collected: (i) for DNA analysis, 2 ml of culture were collected daily, centrifuged at 7267 × g for 10 min, and pellets were stored at −80°C; (ii) for RNA analysis, 5 ml of culture were obtained daily during steady-state and centrifuged at 2268 × g, 4°C, for 15 min. Pellets were resuspended in 500 μL of RNAprotect (Qiagen), and stored at −80°C; and (iii) for spent medium analysis, the supernatants from (ii) were filter-sterilized and stored at −80°C.

The establishment of the community was shown to be reproducible, as demonstrated by repeating the experiment initiated with 5% serum and shifted to 50% serum in two independent runs. Community composition assessed for 26 consecutive days by 16S rRNA gene sequencing showed consistent daily patterns between runs (Supplementary Fig. S2a and b). Day-to-day variation in species relative abundances was strongly correlated between runs, with 17 of 22 species exhibiting Spearman correlation coefficients greater than 0.7 (Supplementary Fig. S2c). Principal coordinate analysis (PCoA) of Bray–Curtis beta-diversity further confirmed reproducibility, showing no significant differences, at the same serum concentration, between runs (PERMANOVA, *P* > .05; Supplementary Fig. S2d).

### Bacterial load determination

Genomic DNA was extracted from culture samples as previously described [6]. Briefly, samples were treated with lysozyme and proteinase K, followed by purification using the DNeasy Blood and Tissue kit (Qiagen). Total bacterial load was quantified by qPCR using universal 16S rRNA primers (5′-TCCTACGGGAGGCAGCAGT-3′ and 5′-GGACTACCAGGGTATCTAATCCTGTT-3′ and the probe, 5′-CGTATTACCGCGGCTGCTGGCAC-3′) as previously described [27].

### Evaluation of community composition via 16S rRNA gene sequencing

Community composition was evaluated by sequencing the V1-V2 regions of the 16S rRNA gene, capable of discriminating among all species in the model [28], using primers 8F (5′-AGAGTTTGATCMTGGCTCAG-3′) and 361R (5′-CYIACTGCTGCCTCCCGTAG-3′) which incorporated MiSeq System (Illumina) adapter sequences and single-end barcodes, as previously described [28, 29]. Amplicon libraries were pooled and sequenced on a MiSeq System (Illumina) using a MiSeq Reagent Kit v3 (2 × 300 bp). Sequences are available at the Short Reads Archive (PRJNA1336263).

### 16S rRNA amplicon data processing and analysis

Raw sequences were processed using Mothur (v1.44.3) [30]. Paired-end reads were first merged into contigs, and contigs containing ambiguous bases, shorter than 150 bp, or with homopolymer stretches exceeding 10 bases were removed. Chimeric sequences were filtered out using UCHIME [31]. After quality control, a total of 7 596 297 sequences were retained (mean ± SD per sample 75 211 ± 48 252) for downstream analysis. Taxonomic classification was performed in Mothur using the k-nearest neighbor (knn) algorithm with k = 1, based on BLAST alignment to a curated reference set from the Human Oral Microbiome Database (v15.1) [32]. To account for variation in 16S rRNA gene copy number across species, the obtained read counts were normalized using species specific copy number information obtained from the PICRUSt2 SC database [33].

Alpha diversity was quantified using the Shannon Index. Beta diversity was evaluated based on Bray–Curtis dissimilarity calculated on species relative abundances and visualized using principal coordinate analysis (PCoA). To identify species most strongly associated with the compositional shifts induced by serum, Spearman’s correlations were calculated between the relative abundance of each species and the first two principal coordinates (PCo1 and PCo2). Differential abundances of species across steady-states were assessed using one-way ANOVA performed on log10 transformed relative abundance values and adjusted using Bonferroni correction. A pseudocount of 1 × 10^−6^ was added prior to transformation to avoid undefined values resulting from zeros. Unless otherwise specified, statistical analyses and ordination visualizations were performed using MATLAB (R2021a), with additional plotting generated using GraphPad Prism (v10).

### Evaluation of community transcriptional activity via metatranscriptomics

Three samples from steady-state in each run and condition were subjected to metatranscriptomic sequencing. RNA was isolated using the RNeasy Mini Kit (Qiagen) following the manufacturer’s protocol with two modifications. First, pellets were resuspended in RLT buffer with β-mercaptoethanol and transferred to silica bead tubes (Lysing Matrix B; MP Biomedicals), followed by lysis in a FastPrep-24 instrument for 20 s. Second, after the first RW1 wash step, on-column DNase digestion was performed using the RNase-Free DNase Set (Qiagen). The protocol was then resumed, and RNA was eluted in nuclease-free water.

RNA integrity was assessed with an Agilent Fragment Analyzer. For library preparation, 100 ng of total RNA per sample were processed with the RiboZero Total Stranded RNA Library Prep Kit (Illumina), including ribosomal RNA removal, according to the manufacturer’s protocol. Final libraries were pooled to a concentration of 10 nM determined by the QuantaBio Universal qPCR kit. After final dilution and denaturing, the pooled library was loaded in one lane at a concentration of 175 pM for 50 base pair paired-end sequencing on an NovaSeq 6000 System (Illumina) at the University at Buffalo Genomics and Bioinformatics Core. Sequences are available at the Short Reads Archive (PRJNA1336263).

### Metatranscriptomic data processing and analysis

Raw RNA sequencing reads were processed with Kneaddata (0.12.2) [34] for quality trimming, and filtering, and removal of potential eukaryotic contaminant reads by alignment to the pig genome (Sscrofa11.1) [35] and the horse genome (EquCab3.0) [36], corresponding to the sources of mucin and serum, respectively, as well as to the human reference genome (GRCh37) [37] to exclude potential human derived contamination. After filtering, reads were aligned using Bowtie2 (2.5.4) [38] to a custom database comprising the reference genomes of the 22 species included in the synthetic community, retrieved from the Human Oral Microbiome Database (HOMD, v15.1). Gene counts were extracted using Samtools (v1.21) [39] based on annotated coding sequences and normalized to reads per kilobase (RPK) to account for gene length. Metatranscriptomic sequencing coverage for each species was assessed by calculating the fraction of genes detected for each genome (Supplementary Fig. S3). Genes were considered detected if at least one RNA sequencing read mapped to the corresponding coding region. For functional interpretation, genes were assigned to UniRef90 gene families using DIAMOND (2.0.15) [40] and subsequently aggregated into KEGG orthologies (KOs), KEGG pathways [41], and Gene Ontology (GO) terms [42].

Species composition based on metatranscriptomic data was derived by normalizing read counts to relative abundance, calculated as the proportion of RPK assigned to each species relative to the total RPK within the sample. To evaluate shifts in the composition of metatranscriptomically active species at steady state, Bray–Curtis beta diversity was assessed and visualized with PCoA. Samples obtained on different days under the same serum condition were treated as independent biological replicates. Correlation analysis with the principal coordinates was applied following the same approach used for 16S rRNA gene sequencing data, to identify species most strongly associated with serum-induced shifts. Alterations in the propositions of individual species across steady-states were then evaluated using one-way ANOVA, consistent with the 16S analysis, with Bonferroni correction for multiple comparisons. Community-wide functional analysis was performed on aggregated functional profiles (e.g. UniRef90 gene families, KOs, KEGG pathways, and GO terms) to characterize the overall functional activity of the community, irrespective of species abundances. Beta diversity of these functional profiles was assessed using Bray–Curtis dissimilarity and visualized with PCoA. Differential expression analysis was performed in R using the DESeq2 package [43]. Redundant GO terms were summarized with REVIGO [44].

Because community-level transcriptomic profiles reflect both variation in the abundance of metabolically active species and changes in gene expression within individual species, a complementary species-specific analysis was also performed. In this analysis, the RPK value of each gene was normalized to the total transcriptional output of its corresponding species, thereby controlling for differences in species abundances. This approach enabled the detection of genuine regulatory responses within taxa rather than changes in transcriptional activity solely attributable to shifts in community composition. Differential expression of genes within each species was identified using DESeq2, and differentially expressed biological processes (BPs) were likewise determined for the six species that showed the strongest transcriptional responses to serum, each exhibiting more than 20% of their expressed genes differentially regulated at the highest serum concentration.

### Metabolomics of spent media

Spent medium samples collected and stored as described above were used for metabolomic analysis performed at the Southeast Center for Integrated Metabolomics (SECIM), University of Florida Health. Samples underwent protein precipitation, followed by collection and drying of the supernatant. Dried extracts were reconstituted for analysis in both positive and negative ionization modes, each run as separate injections. Global metabolomics profiling was carried out on a Thermo Q-Exactive Orbitrap mass spectrometer equipped with a Dionex UHPLC and autosampler. All samples were analyzed under heated electrospray ionization (positive and negative) at a mass resolution of 35 000 (m/z 200). Separation was achieved on an ACE 18-pfp column (100 × 2.1 mm, 2 μm) with mobile phase A (0.1% formic acid in water) and mobile phase B (acetonitrile), a flow rate of 350 μL/min, and a column temperature of 25°C. Injection volumes were 4 μL for negative ions and 2 μL for positive ions. Each batch included extraction blanks, quality controls, and samples. Internal and injection standards were added to all samples to monitor batch reproducibility.

Data from positive and negative ionization modes were analyzed separately. A total of 1663 features were detected in positive mode and 2323 in negative mode. Feature detection, deisotoping, alignment, and gap filling were performed in MZmine [45]. Adducts and complexes were identified and removed. Features were annotated by searching against the SECIM’s internal retention time library of ~1100 standards. Features lacking confident annotation were further queried against the Human Metabolome Database [46] to assign putative identities based on m/z, ionization mode, and molecular weight tolerance (±5 ppm). LC–MS metabolomics data have been deposited in the Metabolomics Workbench under accession number ST004636.

Processed data were imported into MetaboAnalyst 5.0 [47] for statistical analysis. Features with >80% missing values were removed, and remaining missing values were imputed with the feature mean. Data were filtered by relative standard deviation (RSD = SD/mean), normalized by sum, log-transformed, and autoscaled using the default settings. Differential features across serum conditions in both ionization modes were identified in MetaboAnalyst using one-way ANOVA with Bonferroni correction for multiple testing. Based on the normalized metabolite abundances, community-level metabolic differences among the three serum conditions (0%, 5%, and 50%) were evaluated by principal component analysis (PCA) using Euclidean distances with statistical significance assessed using PERMANOVA followed by Bonferroni post hoc correction.

### Measurement of protein concentration of fresh media

Fresh hog mucin medium was prepared as described above with 0%, 5%, and 50% (v/v) heat-inactivated horse serum. Protein concentrations were quantified using the Pierce BCA Protein Assay Kit (Thermo Fisher Scientific).

### Determination of ammonium/ammonia concentrations in spent media

The concentration of ammonium/ammonia in spent media was quantified using a colorimetric assay kit (Abcam). Samples were diluted as required to ensure their values fell within the linear range of the standard curve, which was generated using an ammonium chloride standard solution.

### Growth of *F. nucleatum* and *P. gingivalis* as monocultures under different serum concentrations

To evaluate the effect of serum on *F. nucleatum* and *P. gingivalis*, both species were cultured in mucin medium, prepared as described above, supplemented with 0%, 5%, or 50% (v/v) heat-inactivated horse serum. The pH of the medium was adjusted to 7.0 before inoculation. Each species was inoculated at a density of 10^7^ cells ml^−1^ and incubated anaerobically at 37°C for seven days. Cultures were sampled daily inside the anaerobic chamber. Biomass of each species was quantified via qPCR, and the pH of culture media was measured using a pH meter (Accumet AB15, Fisherbrand).

## Results

### Serum promotes greater biomass and inter-microbial assemblage formation

We developed a synthetic polymicrobial community model in continuous culture consisting of 22 prevalent species of subgingival plaque (Fig. 1a), selected based on previous human studies [5]. The synthetic community was established under controlled conditions, including a consistent pH of 7.15 ± 0.15, a microaerophilic atmosphere (2% O_2_), and a mucin-based basal medium [25], to replicate the oral environment, as nutritional source. Heat-inactivated serum, to a final concentration of 5% or 50% by volume, was introduced during initial community assembly or at steady-state to assess the impact of this nutritional substrate on the community properties (Fig. 1b).

**Figure 2:**
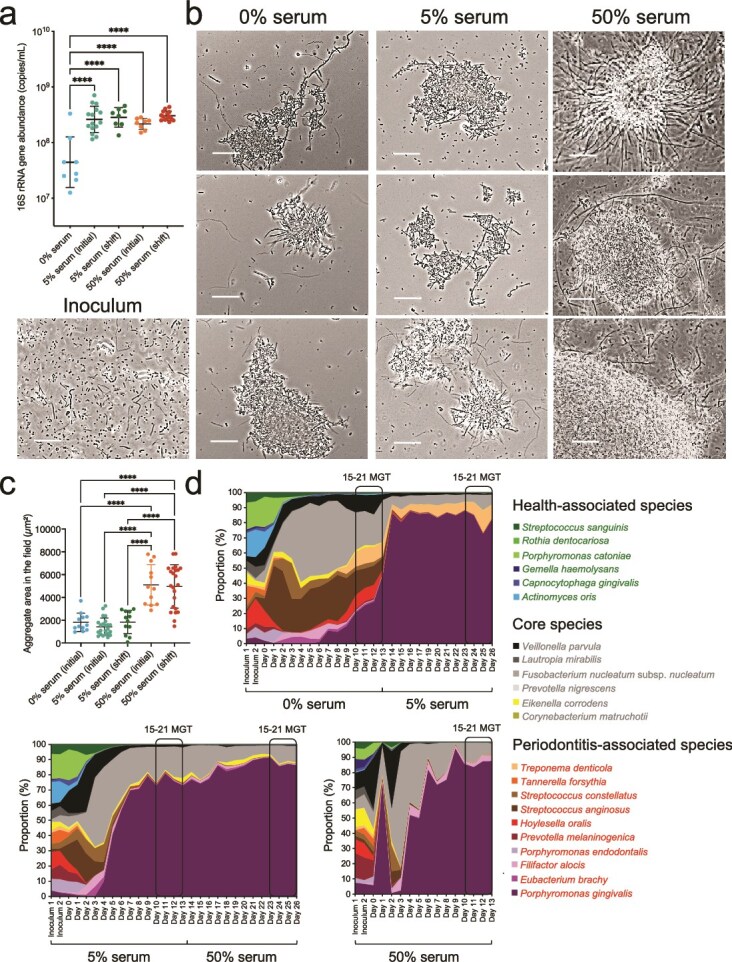
Effect of serum on community biomass, microbial assemblages and species abundances. (a) Total bacteria load at each steady state quantified using qPCR. (b) Phase-contrast micrographs of steady-state cultures showing microbial aggregate formation under different serum concentrations. All scale bars represent 20 μm. (c) Quantification of the area occupied in micrographs by polymicrobial aggregates at steady state under different serum concentrations. Aggregate area was measured from microscopic images using ImageJ. (d). Community taxonomic composition during the assembly period and theoretical steady state phases (15–21 mean generation times, MGT) as characterized via 16S rRNA sequencing. For panels (a) and (c) statistical significance was determined using one-way ANOVA with Bonferroni post hoc correction (^****^ = *P* < .0001).

Given that gingival inflammation and exposure of subgingival microbiome communities to a higher flow rate of GCF are associated with increased biomass [6], we first evaluated the effect of serum on total bacterial load. We found that serum, regardless of concentration, significantly increased, by ~10-fold, the biomass compared to serum-free conditions (Fig. 2a). We next performed microscopic examination of fresh planktonic culture samples to determine whether serum influenced the physical organization and appearance of microbial cells. Contrary to the inocula which contained single cells, chemostat cultures consisted of microbial aggregates comprised of cells with diverse morphologies (Fig. 2b). These planktonic aggregates occurred in the absence and presence of serum and were reminiscent of the multi-species clusters that commonly occur in the planktonic salivary phase within the oral cavity [48]. Although total load was similar between 5% and 50% serum (Fig. 2a), large dense aggregates characterized communities in the highest serum concentration (Fig. 2b and c), suggesting that an excess of serum components enhances physical interactions among subgingival bacteria promoting the formation of structured microbial assemblages.

### Serum affects polymicrobial community structure

To monitor daily community composition, we employed 16S rRNA gene sequencing (Supplementary Table S3). Species distributions in the inoculum were relatively even (Fig. 2d and Supplementary Fig. S4). However, when growing in the basal mucin medium (no serum), community evenness dropped during the first 4 days in culture, with 11 species maintaining a relative abundance at or above 1% dominating the community for the following days until steady-state. These species were core species or periodontitis-associated taxa, whereas health-associated commensals were present but in lower abundance (Supplementary Table S3). Introduction of serum, at any concentration, and either during initial community assembly or at steady-state, affected community structure further decreasing evenness and promoting the dominance of the periodontitis-associated species *Porphyromonas gingivalis*, only consistently accompanied across serum conditions by *Fusobacterium nucleatum* subsp. *nucleatum*, in moderate abundance.

**Figure 3:**
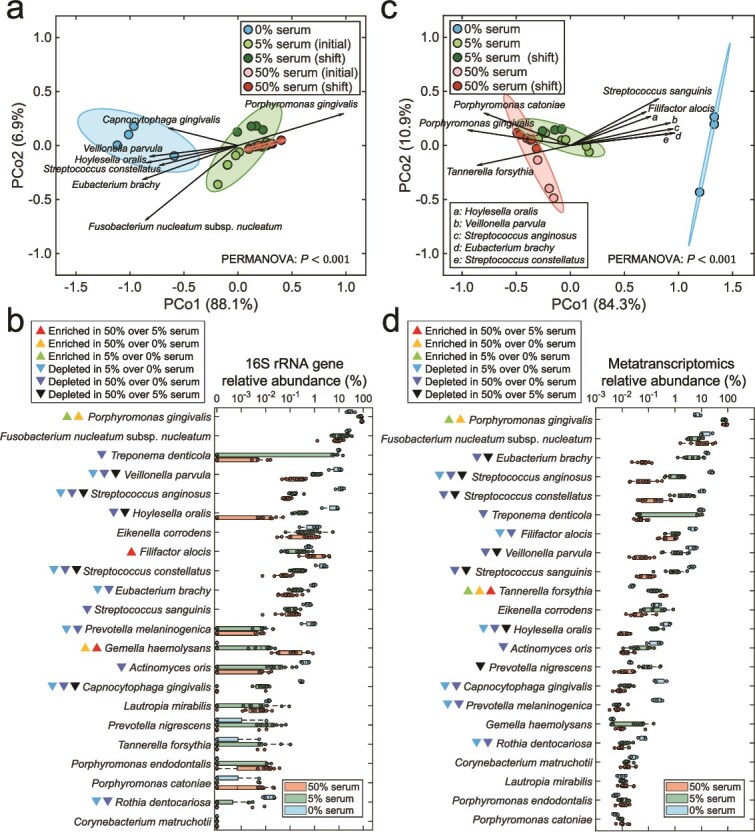
Effect of serum on steady-state microbial community composition, as analyzed through 16S rRNA sequencing and metatranscriptomics. (a) Principal coordinate analysis (PCoA) of Bray–Curtis beta-diversity based on 16S rRNA data across different serum concentrations at steady state. Group-level differences were assessed with PERMANOVA test with Bonferroni correction as post hoc analysis. *P* values: 0.094 for 5% serum (initial) vs. 5% serum (shift); 1 for 50% serum (initial) vs. 50% serum (shift); 0.002 for 0% serum vs. 5% serum; 0.001 for 0% serum vs. 50% serum; 0.011 for 5% serum vs. 50% serum. Arrows indicate species significantly correlated with PCo1 (*P* < .05), with arrow direction and length reflecting Spearman’s correlation coefficients between species relative abundances and the PCoA axes. (b) Box plots of 16S rRNA relative abundances across serum concentrations. Significance of differences was determined using the one-way ANOVA with Bonferroni correction; colored triangles denote differentially enriched or depleted species. (c) PCoA of Bray–Curtis beta-diversity based on steady-state metatranscriptomic species relative abundances across serum concentrations. PERMANOVA *P* values: 0.318 for 5% serum (initial) vs. 5% serum (shift); 0.117 for 50% serum (initial) vs. 50% serum (shift); 0.012 for 0% serum vs. 5% serum; 0.014 for 0% serum vs. 50% serum; 0.005 for 5% serum vs. 50% serum. Arrows represent species significantly correlated with PCo1 (*P* < .05) by Spearman’s correlation. (d) Box plots of species relative abundances across serum concentrations based on metatranscriptomics. Statistical significance was determined using the one-way ANOVA with Bonferroni correction; colored triangles denote differentially enriched or depleted species.

A comparison of steady-state community structure via Bray-Curtis distances revealed distinct clustering between serum-free and serum-supplemented conditions (Fig. 3a, PERMANOVA, *P* = .002 for 0% vs. 5% serum and *P* = .001 for 0% vs. 50% serum). The timing of serum addition, whether during assembly or at steady-state, did not have a significant effect on community structure. However, communities grown at different serum concentrations (5% vs. 50%) showed statistically significant differences in composition (PERMANOVA, *P* = .02), although clusters overlapped suggesting a lower effect size than when comparing these groups to the no-serum condition (Fig. 3a). An analysis of the species driving these shifts showed that *P. gingivalis* was the main beneficiary of having serum in the growth medium, at the expense of depletion of certain core and periodontitis-associated species (Fig. 3a). Additionally, analysis of changes in proportions of individual species showed that *P. gingivalis* was the only species enriched in 5% serum when compared to no serum, with this enrichment remaining stable in 50% serum. A higher serum concentration (50%), however, supported higher proportions of the periodontitis-associated species *Filifactor alocis*, and of the health-associated *Gemella haemolysans*. Most other species were depleted under serum (Fig. 3b).

Because 16S rRNA gene sequencing does not distinguish between metabolically active and inactive species, we performed metatranscriptomic sequencing to evaluate transcriptionally active community members at steady-state (Fig. 3c). This analysis confirmed the global-scale compositional shifts previously observed via 16S rRNA gene sequencing. However, in contrast to the 16S rRNA gene based analysis, in which *P. gingivalis* was the primary beneficiary of serum introduction, the metatranscriptomic analysis indicated that additional *Bacteroidota* taxa, including *T. forsythia* and *Porphyromonas catoniae*, were associated with the transition from no serum to serum. Figure 3d shows levels for transcriptionally active individual species across conditions showing that all 22 species were detected at steady-state, demonstrating the model successfully sustains a diverse and functionally active microbial community. Although *T. forsythia*, a species strongly associated with periodontitis, exhibited low abundance by 16S rRNA gene sequencing, it was moderately abundant in the metatranscriptomics data, with its transcriptional activity increasing proportionally to serum concentration. Other species showed comparable trends between the metatranscriptomics and 16S rRNA gene sequencing data, with most species decreasing in abundance in the presence of serum. *F. nucleatum*, an abundant component of dental plaque in both health and periodontitis did not change its relative abundance upon serum addition according to both 16S and RNA sequencing, consistent with its role as a core subgingival species. Evaluation of changes in sum abundances for health-associated, core and periodontitis-associated species showed that indeed serum favored the periodontitis-associated group at the expense of health-associated taxa (Supplementary Fig. S5). Overall, these taxonomic analyses show that the community growing without serum was more diverse and dominated by core species and periodontitis-associated taxa, whereas introduction of serum as a nutrient source changed diversity and community structure promoting the competitive dominance of *P. gingivalis*.

### Serum promoted community-wide metabolic shifts increasing alkaline and decreasing acidic end-products

**Figure 4:**
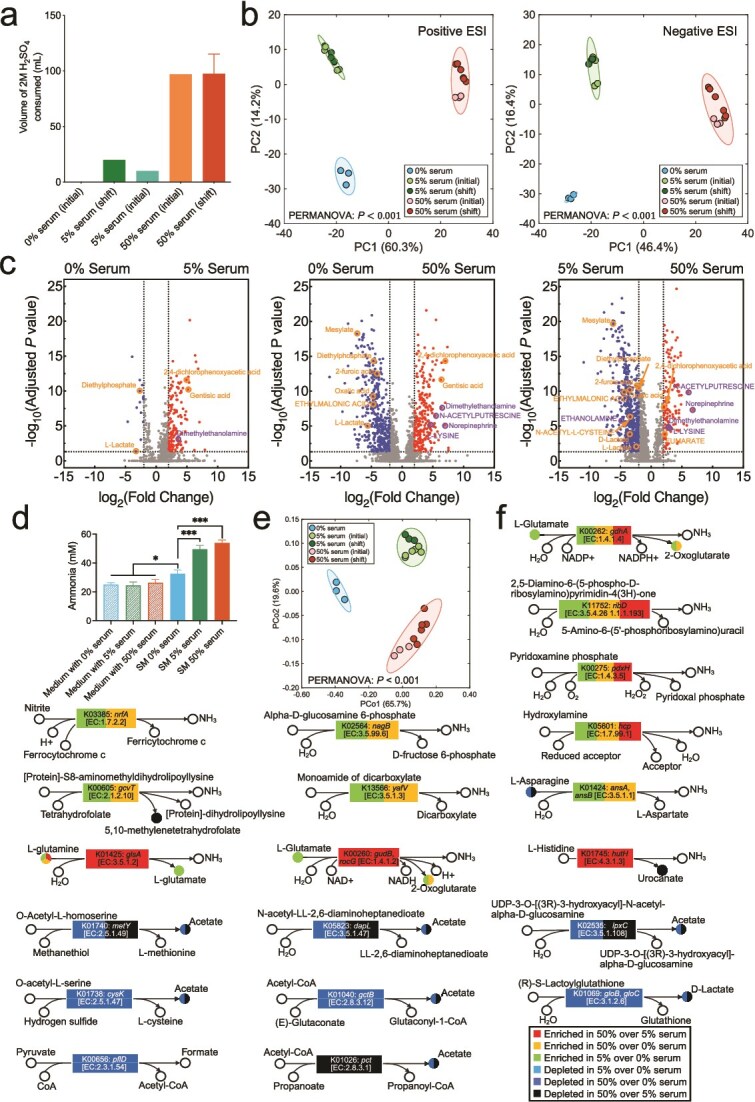
Effect of serum concentration on community metabolism, end-products and environmental pH. (a) Acid consumption, per day, at steady state in the chemostat system for each serum concentration. (b) Principal component analysis (PCA) of metabolomic peaks (positive and negative ESI modes) across steady-state samples with different serum concentrations. PERMANOVA tests were used to evaluate group-level differences, with Bonferroni correction. *P* values: .005 for 0% vs. 5% serum; 0.011 for 0% vs. 50% serum; *P* < .001 for 5% vs. 50% serum in positive ESI mode; and 0.013 for 0% vs. 5% serum; 0.011 for 0% vs. 50% serum; *P* < .001 for 5% vs. 50% serum in negative ESI mode. (c) Volcano plots of metabolites detected by LC–MS showing differential abundance across serum concentrations, identified using one-way ANOVA with Bonferroni correction. Red dots indicate metabolites statistically significantly enriched in higher serum, and blue dots indicate metabolites depleted. Acidic products (pKa < 4) are highlighted in orange and basic products (pKa > 9) in purple. Metabolites annotated using a reference standards database are shown in uppercase, whereas those queried against the human metabolome database (HMDB) are shown in lowercase. Group-level differences were assessed using PERMANOVA with Bonferroni correction. (d) Bar plot of ammonia concentrations measured by a colorimetric assay in uninoculated medium and spent media (SM) from steady state cultures. Significance of differences between conditions was evaluated using one-way ANOVA with Bonferroni correction (^*^ = *P* < .05, ^***^ = *P* < .001). (e) Principal coordinate analysis (PCoA) of metatranscriptomic KEGG orthologies (KOs) based on Bray-Curtis distances for steady-state samples. Group-level differences were assessed using PERMANOVA with Bonferroni correction. *P* values: .006 for 0% vs. 5% serum, .007 for 0% vs. 50% serum, and *P* < .001 for 5% vs. 50% serum. (f) KOs involved in ammonia production and upregulated by serum or in acidic product formation and downregulated by serum. Differentially expressed KOs (rectangles) were identified by DESeq2 analysis of metatranscriptomic data, and corresponding enriched or depleted metabolites (circles) were derived from the LC–MS analysis shown in panel c. Colors denote the direction of change across serum concentration comparisons.

We observed a notable increase in acid consumption in the chemostat under serum, especially at 50% (Fig. 4a). Given that the basal pH of different media did not differ and that the system was designed to maintain a pH of 7.15 ± 0.15, this increase in acid utilization suggested a metabolic shift towards production of less acidic and more alkaline fermentation end-products. To investigate this, we performed LC–MS analysis of spent media from steady-state cultures. Analysis of global metabolomic profiles showed the no serum, 5%, and 50% serum communities as distinct (Fig. 4b). Differential metabolite abundance analysis identified a small number of metabolites changed in the comparison of spent media from no serum versus 5% and a larger number showed differences in the 5 to 50% serum comparison (Fig. 4c). This analysis showed that strong acidic end-products such as lactate, 2-furoic acid, mesylate, and oxalic acid were depleted in conditions with serum, whereas basic and amine-containing metabolites, including dimethylethanolamine, N-acetylputrescine, and l-lysine were enriched. Detailed differential metabolites are provided in Supplementary Table S4. Because the LC–MS technique used did not allow direct measurement of ammonia, a key alkaline by-product of amino acid metabolism, we next employed a colorimetric assay to quantify ammonia concentrations in the spent media. This analysis showed that ammonia levels were significantly elevated in steady-state communities grown with serum (5% and 50%) compared to serum-free conditions, with a trend towards higher levels at 50% serum (Fig. 4d). In summary, these results suggest serum induces metabolic shifts increasing ammonia and other basic metabolites and depleting acidic end-products.

**Figure 5:**
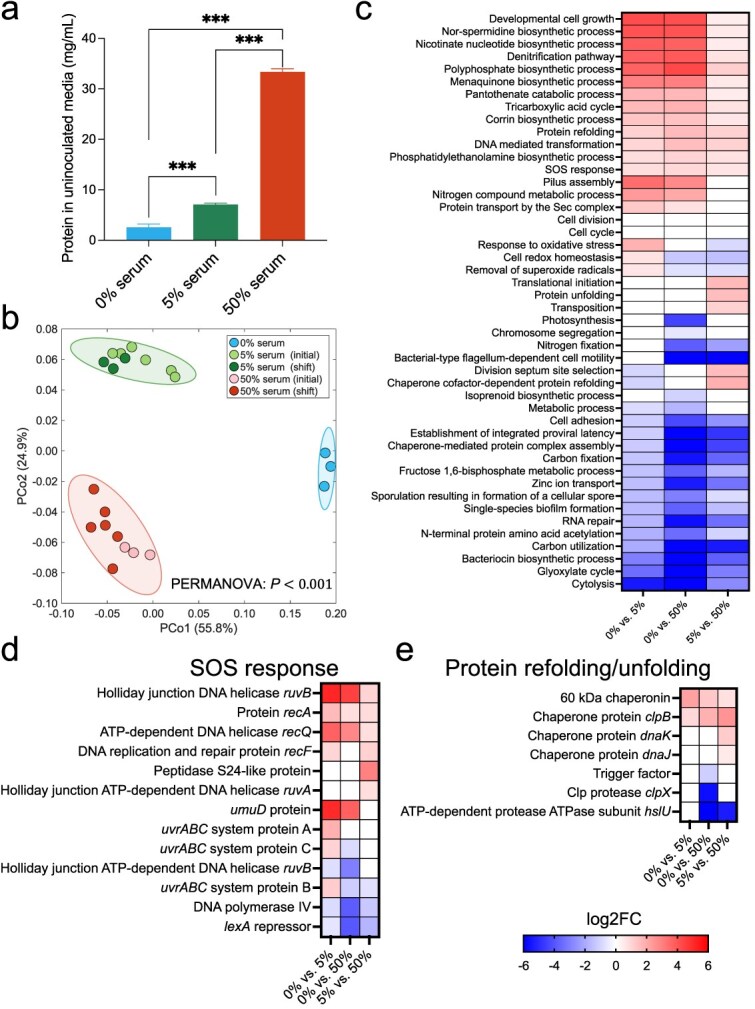
Effect of serum concentration on community-wide transcriptomic activities. (a) Bar plot showing the total protein content of uninoculated media with varying serum concentrations. Significance of differences was evaluated using one-way ANOVA with Bonferroni correction (^***^ = *P* < .001). (b) Principal coordinate analysis (PCoA) of Bray–Curtis beta-diversity based on metatranscriptomic data grouped by gene ontology (GO) terms across different serum concentrations. Group-level differences were assessed using PERMANOVA test with Bonferroni correction. *P* values: .017 for 0% vs. 5% serum, .005 for 0% vs. 50% serum, and .001 for 5% vs. 50% serum. (c) Heatmap of log2 fold-changes (log2FC) of differentially expressed biological processes (BPs) across serum concentrations. (d–e) Differentially expressed UniRef90 gene families involved in (d) SOS response, and (e) protein refolding/unfolding. Differential expression across panels (c–e) was determined using DESeq2 (log2FC > 0.3785 or < −0.3785, FDR < 0.05).

The shifts in metabolite profiles with serum suggested a potential change in transcriptomic activity related to metabolism. To explore this further, community-wide metatranscriptomic reads (not binned by species) were annotated using KEGG orthologies (KOs). Principal Coordinate Analysis revealed distinct clustering of communities growing in no serum, 5% and 50%, confirming serum altered the community-wide metabolic activities in a concentration dependent manner (Fig. 4e). Among KOs upregulated by serum were several genes involved in reactions that result in production of ammonia, whereas KOs associated with acidic end-product formation were downregulated (Fig. 4f). Genes critical to nitrite reduction, such as K03385 (*nrfA*, nitric oxide reducatse), and nitrosative stress protection, including K05601 (*hcp*, hybrid cluster protein), were significantly upregulated by serum, as were genes involved in glutamine and histidine catabolism, all of which contribute to ammonia production. In contrast, genes responsible for acetate and lactate production were downregulated in response to serum, with metabolomics confirming depletion of these end-products in spent media (Fig. 4f). Detailed differential KOs are provided in Supplementary Table S5. Furthermore, an analysis of KEGG pathways showed that serum was associated with enrichment of nitrogen metabolism, partly through upregulation of amino acid degradation pathways, including histidine metabolism (Supplementary Fig. S6). Because these results suggested increase protein catabolism under serum, we next measured the total protein content of the uninoculated media used in the three conditions tested, which showed 5% and 50% serum had ~2-fold and ~12-fold more protein, respectively, than the no serum medium (Fig. 5a) confirming serum increased the availability of protein substrates to be utilized by the community, which in turn was enriched for genes related to protein catabolism. Serum was also associated with an increase in glycosaminoglycan and other glycan degradation suggesting the community could utilize these components of serum (Supplementary Fig. S6). Collectively, these findings confirm serum induces a metabolic shift favoring protein and nitrogen metabolism and ammonia production, whereas suppressing acidic end-product formation.

### Serum modulates transcriptomic activities inducing a stress response

To gain broader insights into functional changes beyond metabolic pathways, we additionally annotated the metatranscriptomic data using Gene Ontology (GO) terms, which capture broader functional attributes not fully represented in pathway based frameworks. Principal coordinate analysis (PCoA) of the metatranscriptomic GO profiles showed significant differences between the no-serum, 5% and 50% serum conditions, again confirming serum influenced community-wide gene expression in a concentration dependent manner (Fig. 5b). Differential expression analysis identified several biological processes (BPs) that varied across serum concentrations (Fig. 5c). Serum promoted the upregulation of the SOS response and protein refolding/unfolding pathways, indicating the community was responding to a stress. Genes involved in DNA repair, such as *recA*, *recQ*, *recF*, *ruvA*, *ruvB*, and the *uvrABC* system, were significantly upregulated, whereas the critical SOS repressor *lexA* was downregulated, pointing to a response to DNA damage (Fig. 5d). Additionally, several chaperones involved in protein maintenance, such as *clpB* and *dnaK*, were enriched in serum, reflecting increased proteostasis activity in response to stress (Fig. 5e).

The denitrification pathway was upregulated in response to serum (Fig. 5c). Serum is known to have nitrate and nitrite at concentrations in the μM range [49, 50], and higher levels of *nrfA*, a key enzyme in nitrite ammonification, were shown to be induced in the community by serum, suggesting an increase in nitrite respiration (Fig. 4f). Production of nitric oxide and reactive nitrogen species (RNS) during nitrite respiration could be, however, a source of DNA and protein damage, potentially explaining the observed upregulation in protective mechanisms, such as DNA repair and protein refolding. When considering other possible sources of DNA and protein damage, such as oxygen, we observed that only a small number of genes directly involved in oxidative stress were upregulated under 5% serum and most oxidative stress genes were actually downregulated under 50% serum (Supplementary Fig. S7). The downregulation of oxidative stress response aligns with a very reduced redox potential (*Eh*) in cultures under all conditions with serum (Supplementary Fig. S8), despite the constant incoming flow of oxygen into the chemostat. Therefore, nitrosative stress seemed a plausible form of stress under serum.

Another GO biological process upregulated by 50% serum was transposition (Fig. 5c), driven by the enrichment of several transposase and recombinase genes (Supplementary Table S6). This response may be connected to stress, because higher ability to mobilize transposable elements could support genetic diversification of the population increasing chances of survival. Other GO biological processes upregulated included cell growth, cell cycle, cell division and translation initiation (Fig. 5c), consistent with the higher biomass of communities under serum. Corrin biosynthesis, which forms the core for corrinoid cofactors such as cobalamin, was also upregulated under serum (Fig. 5c).

### Serum induces species-specific changes in transcriptomic activity

**Figure 6:**
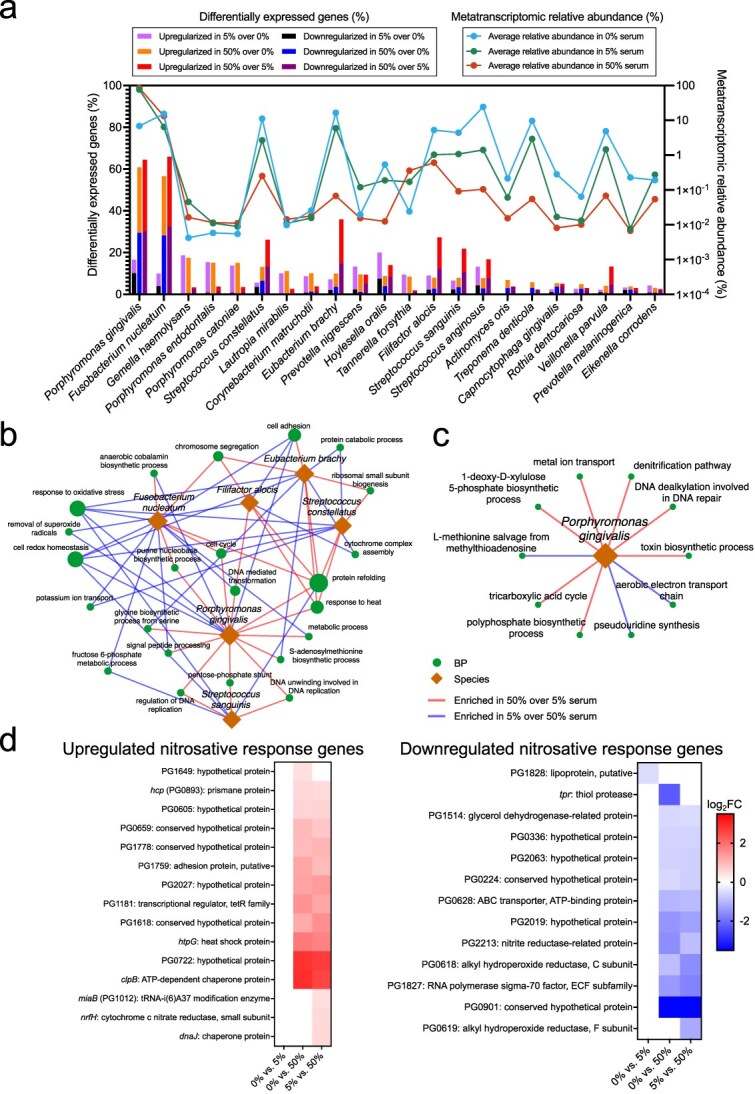
Changes in metatranscriptomic activities within species when growing as part of the 22 spp. community under different serum concentrations. (a) Bar plot showing the percentage of differentially expressed genes relative to the total number of genes detected per species, comparing steady states across serum concentrations. Lines represent the average metatranscriptomic relative abundance of each species across serum conditions. (b) Network of biological processes (BPs) enriched or depleted in 5% vs. 50% serum in the six species most responsive to serum. Diamonds represent species, and circles represent BPs, with edges connecting species and BPs enriched in either 5% (blue) or 50% serum (red). Only BPs with node degree >2 are shown, with node size proportional to degree. (c) Network of BPs uniquely enriched or depleted in *Porphyromonas gingivalis* under 5% vs. 50% serum (d) Heatmaps showing up- and down-regulated genes part of the response to nitrosative stress in *P. gingivalis*. The set of nitrosative stress response genes were identified by previous in vitro studies (Belvin et al 2019 [53], Lewis et al 2012 [54]). Differential expression across panels (a–d) was determined using DESeq2 (log2FC > 0.3785 or < −0.3785, FDR < 0.05).

We investigated species-specific transcriptomic responses to serum by normalizing gene expression within each species to its total transcriptomic output. This allowed identification of differentially regulated genes within each species, independent of changes in community composition across conditions. Six species, the core species *F. nucleatum*, the periodontitis-associated *P. gingivalis*, *Streptococcus constellatus*, *Eubacterium brachy*, and *F. alocis*, and the health-associated commensal *Streptococcus sanguinis*, exhibited the most substantial transcriptomic shifts, with over 20% of their expressed genes differentially regulated in response to higher serum levels (Fig. 6a).

We analyzed the biological processes affected in the most serum-responsive species (Supplementary Fig. S9). Biological processes enriched or depleted in the comparison between 5% and 50% serum were identified across multiple species (Fig. 6b). Protein refolding was upregulated in 5 of the 6 species analyzed, confirming our findings of a community-wide response to maintain protein homeostasis at higher serum concentrations. Conversely, oxidative stress responses and cell redox homeostasis processes were downregulated, also confirming community-wide findings. Among other BPs downregulated in response to serum in several species was cell adhesion, with *P. gingivalis* downregulating its adhesion protein and virulence factor fimbrillin (*fimA*), which has been previously shown to decrease in response to serum and saliva [51]. In addition, a BP downregulated in several species by higher serum was potassium transport. This function has been shown to be downregulated in human subgingival communities during periodontitis [52].

Individual species also exhibited distinct responses to higher serum levels (Fig. S7, Supplementary Tables S7–S12). For instance, *P. gingivalis* uniquely upregulated genes related to nitrite metabolism, which could serve as precursor for RNSs (Fig. 6c). *nrfH*, a membrane-bound cytochrome c which acts as a redox partner to the nitrite reductase *nrfA*, was upregulated*.* Additionally, *P. gingivalis* upregulated DNA repair (Fig. 6c). Given that *P. gingivalis* was the most abundant species in both 16S rRNA and metatranscriptomic analyses, this species may play a pivotal role in driving nitric oxide and RNS release.

Previous in vitro studies have identified several genes in *P. gingivalis* as differentially regulated when growing in nitrite [53, 54]. Comparison with these studies revealed that many previously identified nitrite-responsive genes were likewise differentially expressed in *P. gingivalis* in our community model in response to serum (Fig. 6d and Supplementary Fig. S10). This included upregulation of major nitrosative stress response genes such as *hcp* (PG0893), *nrfH*, and *clpB*. This result strongly supports the hypothesis that nitrosative stress plays a key role in modulating transcriptomic activity in this community model under higher serum conditions.

Additional species-specific responses to higher serum were also observed. In *P. gingivalis*, the GO biological process S-adenosylmethionine biosynthesis was upregulated, and metK, the gene encoding the enzyme responsible for S-adenosylmethionine (SAM) synthesis, showed increased expression under higher serum in both *P. gingivalis* and *F. alocis* (Supplementary Tables S7 and S11). SAM has been shown to participate in a variety of cellular processes including DNA repair, but also in cell growth, survival and quorum sensing [55–58]. The core species *F. nucleatum* showed a strong upregulation of nickel transport under 50% serum suggesting this metal ion is important for its survival in this environment. In contrast, the commensal *S. sanguinis* upregulated, under 50% serum, ABC transporters for glycine betaine, which acts as a protectant against osmotic stress in bacteria [59], suggesting the high protein levels under high serum may have created colloid osmotic pressures harmful for this species.

### Species-specific transcriptomic alterations in *P. gingivalis* explain increased pH under serum

**Figure 7:**
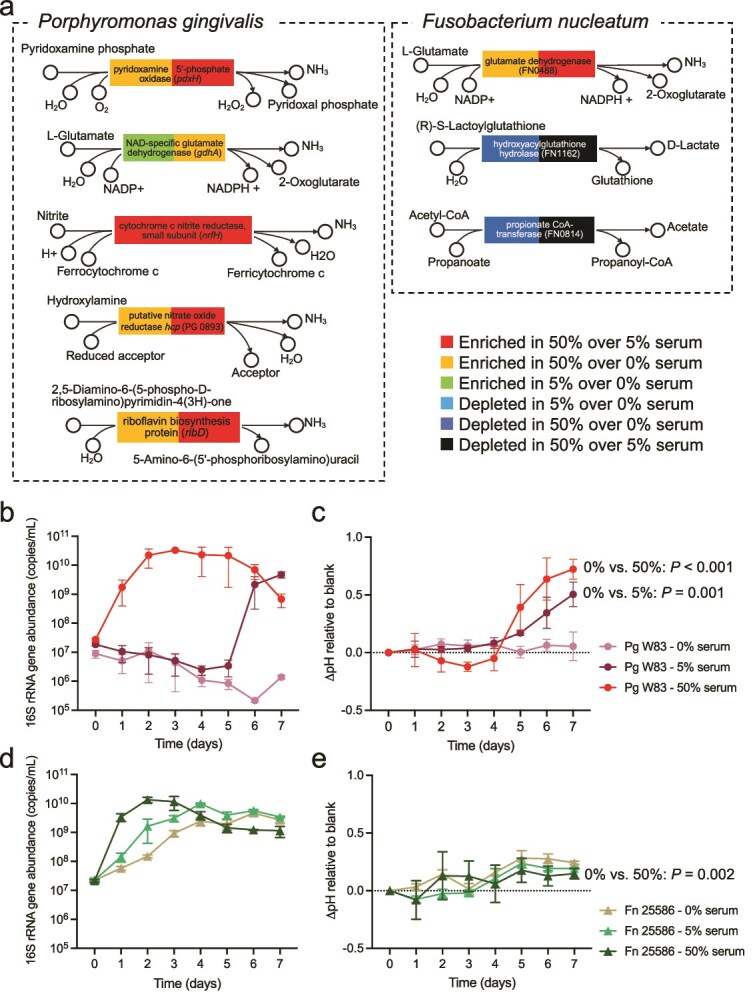
Effect of serum on the metabolic activities, growth and ability to modify the pH of *Porphyromonas gingivalis* and *Fusobacterium nucleatum*. (a) Differentially expressed genes involved in ammonia and acidic end-product formation in *P. gingivalis* and *F. nucleatum* as measured in steady-state chemostat cultures. (b) Biomass of *P. gingivalis* in batch monoculture under varying serum concentrations. (c) pH change (ΔpH) relative to fresh medium for *P. gingivalis* cultured in varying serum concentrations. (d) Biomass of *F. nucleatum* cultured under varying serum concentrations. (e) pH changes (ΔpH) relative to fresh medium for *F. nucleatum* cultures under varying serum concentrations. Statistical significance for ΔpH (panels c and e) was assessed on day 7 using one-way ANOVA with Bonferroni post hoc correction; only pairs showing significant differences were annotated.

Given the observed community-wide changes in KOs associated with increased ammonia production and reduced acidic byproducts, we investigated significantly regulated genes that could potentially affect the environmental pH in *P. gingivalis* and *F. nucleatum*, the two most abundant species in the community. Genes resulting in ammonia production, such as *pdxH*, *nrfH*, PG_0893 (*hcp*), and *ribD*, were upregulated in *P. gingivalis* in response to serum (Fig. 7a). In contrast, *F. nucleatum* exhibited upregulation of FN0488 (glutamate dehydrogenase), whereas genes involved in acid production, such as FN1162 (hydroxyacylglutathione hydrolase) and FN0814 (propionate CoA-transferase), were downregulated. These findings point to the specific reactions in these microorganisms capable of affecting the environmental pH, with *P. gingivalis* showing a plethora of ammonia producing reactions upregulated by serum.

To validate the role of serum in promoting pH-modulating metabolism in *P. gingivalis* and *F. nucleatum*, we conducted culture experiments to assess how varying serum concentrations affect their growth and capacity to alter environmental pH. Fresh media incubated under the same conditions was included as the benchmark, and the difference between the culture pH and fresh media was denoted as ΔpH. *P. gingivalis* needed serum in the medium to grow as a monoculture (Fig. 7b), in contrast to its ability to grow as an abundant community member in the 22-species community with no serum (Fig. 2d). These results, unexpectedly, showed the essential role of inter-species cooperation to support growth of this pathogen in mucin. 50% serum reduced the lag phase of *P. gingivalis* (Fig. 7b), consistent with the advantage gained by *P. gingivalis* in our chemostat community under serum. *F. nucleatum* also benefited from the presence of higher serum, although it was able to grow as a monoculture without it (Fig. 7d). With respect to the ability of these species to modify the pH, only *P. gingivalis*, when growing under serum, significantly increased the ΔpH, whereas *F. nucleatum* only slightly modified the pH towards alkalinity (Fig. 7c, e). Collectively, these findings suggest that *P. gingivalis* is a key driver of the metabolic adaptations observed in the microbial community, contributing significantly to the regulation of environmental pH under serum-enriched conditions. Increased basic metabolite production occurred not only because of *P. gingivalis* enrichement under serum, but due to transcriptional regulation within this species.

## Discussion

In vitro model systems are essential for dissecting the ecological factors that shape microbiome communities [60–62]. Synthetic communities grown in chemostat-based continuous culture systems provide reproducible and tractable laboratory models that allow systematic evaluation of the influence of different ecological variables on community phenotypes. Here, we present a synthetic community comprised of prevalent and abundant human subgingival species that can be used as a model to probe how specific taxa, gene functions, and environmental factors shape community traits. This model follows pioneering work in the pre-omics era that developed defined-inoculum oral communities in chemostats greatly advancing understanding of oral microbial ecology [16, 63]. The current model, however, includes a greater number of species, selected based on current knowledge of the subgingival microbiome, and utilizes “omics” techniques for in-depth community characterization over the longitudinal course of experiments [16, 63].

The community model was established using a mucin-based basal medium to mimic salivary fluid, the primary nutrient source for oral microorganisms. Prior work using this medium has shown that oral bacteria cooperate through complementary glycosidase and protease activities to degrade the mucin substrate, which is able to support growth of a polymicrobial community [64]. Subgingival communities, however, are also continuously bathed by GCF, which flows more rapidly and changes in composition under inflammatory conditions [22, 65, 66]. We used serum as a surrogate as it resembles GCF, at least in terms of its protein composition [67–70]. Serum has been previously utilized as a sole nutrient source for native subgingival communities, with prior studies showing oral microbial consortia readily degrade serum glycoproteins [71]. Both mucin and serum can therefore serve as nutritional sources for oral microbial communities. However, although mucin and serum are both rich in glycoproteins, the nature of these two nutritional substrates is different, as demonstrated here by the inability of *P. gingivalis* to grow in mucin as a monoculture, where it depended on the presence of the community, whereas addition of serum to the mucin medium readily supported monoculture growth of *P. gingivalis*. The preferential utilization of serum by *P. gingivalis* likely underlies its competitive dominance observed in the community when serum became available. Our results therefore show that serum is an essential nutritional substrate for this pathogen, explaining its tropism towards the subgingival sulcus and its enrichment in periodontitis, where it can become a dominant community member reaching abundances up to 34% (Supplementary Table S1). The higher abundances for *P. gingivalis* in our community model could have been the result of the lower diversity in the model compared to subgingival plaque, and to other environmental factors, such as higher oxygen in vivo, which may limit *P. gingivalis* outgrowth.

The community model included species that are abundant members of the human subgingival microbiome in health as well as in periodontitis. Although some species became dominant, all species were transcriptionally active and therefore the model provides a platform to interrogate drivers of shifts in community configuration between healthy- and disease-like states. Our study used this community model to evaluate the influence of nutrients, specifically serum, on the community properties. We were, however, unable to evaluate the influence of serum on a health-like community as the community established in mucin, without serum, resembled a periodontitis-associated community with its composition dominated by a diverse group of core species and pathobionts, whereas health-associated species were present in low abundance. Although we supplied 2% oxygen to the culture, the community readily metabolized it and maintained a very low redox potential, suppressing the growth of facultative health-associated commensals. These results suggest that the nutritional pressure of serum is not required to establish a periodontitis-like community, and other factors such as anaerobiosis may play a more important role to promote pathobiont growth and suppress health-associated species. Our results are comparable to a previous study [12] which enriched natural oral communities in a mucin-based medium, under anaerobiosis, also testing the effect of adding heat-inactivated serum on community composition, finding that the mucin-rich medium supported a community enriched for certain periodontitis-associated bacteria, such as *T. denticola*, with very low levels of health-associated commensals, whereas addition of serum favored the growth of other periodontitis-associated taxa, including *P. gingivalis* and *F. alocis* [12]. Similar to those results, health-associated commensals remained at a very low level in our communities, *T. denticola* was enriched in mucin without serum, and *P. gingivalis* and *F. alocis* were favored by serum. In addition, other in vitro enrichment studies with natural communities have shown that *Treponema* spp. are favoured by low or no serum conditions, whereas *Bacteroidota* are enriched under higher serum levels [13, 24]. These previous studies and our results suggest that serum serves as a nutritional pressure that favors certain periodontitis-associated bacteria but disfavors others, including spirochaetes.

Although serum appears as non-essential for a periodontitis-like community to establish, it changed the community in ways that recapitulate periodontitis-associated dysbiotic shifts, including promotion of higher biomass [5, 6], increasing the environmental pH and changing gene expression in a manner that parallels some of the transcriptomic alterations seen in human periodontitis [52, 72]. Some of these transcriptomic changes included increased amino acid catabolism, especially upregulation of histidine degradation, as well as increased nitric oxidoreductase activity, chaperone and protein folding activities and downregulation of potassium uptake, which have been previously reported as associated with metatranscriptomes of periodontitis sites compared to health [52, 72]. Prior studies also found an increase in the GO term lipid A biosynthesis in periodontitis compared to health [52], with our model finding that serum upregulated lipopolysaccharide biosynthesis in a concentration-dependent manner (Supplementary Fig. S6). Lipopolysaccharides are critical virulence factors for induction of inflammation by the oral microbiome [73]. Furthermore, a study dissecting longitudinal microbiome transcriptomic changes during periodontitis progression recently found that activation of cobalamin contributes to progression [11], whereas we found serum increased biosynthesis of corrin, which forms the structural core of cobalamin. Altogether these results show that although serum as a nutrient source is not necessary for a community with periodontitis-like taxonomic configuration to establish, serum plays a role shaping the community properties increasing its dysbiotic-like features.

Serum was seen to promote a more basic environmental pH due to increased ammonia production and decreased acidic end-products. *P. gingivalis*, the most abundant species under serum, was seen to play a major role increasing the pH as seen from its transcriptomic profile and its ability to increase the pH in batch monoculture. Previous work has shown that *P. gingivalis* can only grow in conditions with pH values between 6.7 and 8.0 [74]. In addition, environmental pH is not only critical for the growth of *P. gingivalis* but also for its virulence as the enzymatic activity of gingipains, the potent cysteine proteinases of *P. gingivalis*, is optimal between a pH of 7.5 and 8.0 [75]. As seen here, *P. gingivalis*, like other microbes, modifies the environmental pH to a favorable one for its own growth. In our chemostat-grown community, we controlled the pH around neutrality, but our results showing increased formation of basic products under serum suggest that in an uncontrolled environment the pH would have increased, potentially influencing the fitness and gene expression of *P. gingivalis* itself and other community members. Indeed, another abundant community member, *F. nucleatum*, can tolerate a wide range of pH values but when cultured at a pH above 8 its phenotype changes with cells becoming significantly elongated, increasing their surface hydrophobicity and biofilm formation [76]. We observed under high serum the presence of very elongated rods, possibly *F. nucleatum*, associated with planktonic microbial aggregates of increased size. Although, the pH of the whole vessel was controlled around neutrality, it is possible that pH gradients existed within these microbial aggregates in which cells exposed to higher pH values changed their phenotype becoming elongated and making them more prone to co-adhesion. The pH of the gingival crevice has been shown to increase from below neutrality in health to values greater than 8 in periodontitis [77], and therefore our results suggest that an increased flow of the serum-like inflammatory exudate in periodontitis and the community activities of *P. gingivalis* contribute to this environmental shift which may shape the community phenotype.

High serum induced protein and DNA repair mechanisms at a community-wide level and when evaluating transcriptional changes within species. By comparing the transcriptomic response of *P. gingivalis* to high serum in our community with previous in vitro investigations of the transcriptional response of monocultures of *P. gingivalis* to nitrite [53, 54], it was clear that there was a significant overlap, and therefore the stress response seen in the community likely resulted from nitrosative stress. Prior studies in humans show upregulation of nitric oxidoreductase activity and proteostasis response genes in periodontitis compared to health and it is therefore likely that in vivo, the serum-like inflammatory exudate is responsible for exerting this pressure on the microbiome [52]. RNS is also a likely stress encountered by oral microorganisms as they leave the mouth and translocate systemically through the circulation [78].

One limitation of our model is the use of laboratory-adapted strains rather than clinical isolates. A synthetic community composed of strains freshly isolated from the same individual may better recapitulate in vivo community configurations and enhance the clinical relevance of the model, because strains coexisting within a host are likely already adapted to one another. Another potential limitation of our model is the use of horse serum, which was selected for feasibility reasons due to the large volumes required for these experiments. Although the impact of horse serum on the model is unclear, the overlap between our findings and results from other in vitro plaque community studies using fetal bovine or human serum, as well as with in vivo metatranscriptomic data, suggests that our model remains biologically relevant.

In conclusion, we present here a synthetic community maintained under a controlled environment in continuous culture in a mucin-rich medium serving as a model of the human subgingival microbiome, which can be used to interrogate the ecological variables that shape community configuration and function. Using this community, we explored the role of serum as an ecological modifier finding that serum increased the community biomass, promoted polymicrobial aggregate formation, increased the environmental pH and induced transcriptional activities that paralleled those of in vivo microbiome communities in periodontitis. Although mucin was sufficient to sustain a periodontitis-like community, addition of serum favored the outgrowth of *P. gingivalis* and modulated the community properties. The serum-like exudate that bathes subgingival communities serves therefore as a nutritional pressure that likely perpetuates dysbiosis.

## Supplementary Material

Supplementary_Table_S1_Species_clin_data_wrag070

Supplementary_Table_S2_Media_and_conditions_wrag070

Supplementary_Table_S3_16S_rRNA_gene_abund_wrag070

Supplementary_Table_S4_Metabolomics_wrag070

Supplementary_Table_S5_Comm_wide_KOs_wrag070

Supplementary_Table_S6_Comm_wide_gene_fam_wrag070

Supplementary_Table_S7_P_gingivalis_wrag070

Supplementary_Table_S8_F_nucleatum_wrag070

Supplementary_Table_S9_S_constellatus_wrag070

Supplementary_Table_S10_E_brachy_wrag070

Supplementary_Table_S11_F_alocis_wrag070

Supplementary_Table_S12_S_sanguinis_wrag070

Li_et_al_Supplementary_Figures_wrag070

## Data Availability

The microbiome sequence data was deposited in the Sequence Reads Archive (accession number PRJNA1336263). The metabolomics data have been deposited in the Metabolomics Workbench under accession ST004636. The custom analysis code used in this study is publicly available on GitHub at https://github.com/liluacrobat/SerumDrivenDysbiosis. Data that support the findings of this study are available from the authors upon reasonable request.

## References

[1] Lloyd-Price J, Arze C, Ananthakrishnan AN. et al. Multi-omics of the gut microbial ecosystem in inflammatory bowel diseases. *Nature.* 2019;569:655–62. 10.1038/s41586-019-1237-9 31142855 PMC6650278

[2] Lee JY, Tsolis RM, Baumler AJ. The microbiome and gut homeostasis. *Science.* 2022;377:eabp9960. 10.1126/science.abp9960 35771903

[3] Usyk M, Carlson L, Schlecht NF. et al. Cervicovaginal microbiome and natural history of *chlamydia trachomatis* in adolescents and young women. *Cell.* 2025;188:1051–1061.e12. 10.1016/j.cell.2024.12.011 39818212 PMC12035847

[4] Abusleme L, Hoare A, Hong BY. et al. Microbial signatures of health, gingivitis, and periodontitis. *Periodontol* 2000;86:57–78. 10.1111/prd.12362 33690899

[5] Diaz PI, Hoare A, Hong BY. Subgingival microbiome shifts and community dynamics in periodontal diseases. *J Calif Dent Assoc* 2016;44:421–35. 10.1080/19424396.2016.12221035 27514154

[6] Abusleme L, Dupuy AK, Dutzan N. et al. The subgingival microbiome in health and periodontitis and its relationship with community biomass and inflammation. *ISME J.* 2013;7:1016–25. 10.1038/ismej.2012.174 23303375 PMC3635234

[7] Griffen AL, Beall CJ, Campbell JH. et al. Distinct and complex bacterial profiles in human periodontitis and health revealed by 16S pyrosequencing. *ISME J* 2012;6:1176–85. 10.1038/ismej.2011.191 22170420 PMC3358035

[8] Lamont RJ, Hajishengallis G. Polymicrobial synergy and dysbiosis in inflammatory disease. *Trends Mol Med* 2015;21:172–83. 10.1016/j.molmed.2014.11.004 25498392 PMC4352384

[9] Hajishengallis G . The inflammophilic character of the periodontitis-associated microbiota. *Mol Oral Microbiol* 2014;29:248–57. 10.1111/omi.12065 24976068 PMC4232466

[10] Li L, Hayashi-Okada Y, Falkner KL. et al. Effect of an intensive antiplaque regimen on microbiome outcomes after nonsurgical periodontal therapy. *J Periodontol* 2025;96:241–54. 10.1002/JPER.24-0141 39925335

[11] Duran-Pinedo A, Solbiati JO, Teles F. et al. Longitudinal host-microbiome dynamics of metatranscription identify hallmarks of progression in periodontitis. *Microbiome.* 2025;13:119. 10.1186/s40168-025-02108-8 40369640 PMC12077055

[12] Naginyte M, Do T, Meade J. et al. Enrichment of periodontal pathogens from the biofilms of healthy adults. *Sci Rep* 2019;9:5491. 10.1038/s41598-019-41882-y 30940882 PMC6445289

[13] Baraniya D, Naginyte M, Chen T. et al. Modeling normal and dysbiotic subgingival microbiomes: effect of nutrients. *J Dent Res* 2020;99:695–702. 10.1177/0022034520902452 31999932 PMC7243421

[14] Ter Steeg PF, Van der Hoeven JS, De Jong MH. et al. Enrichment of subgingival microflora on human serum leading to accumulation of *Bacteroides* species. *Peptostreptococci and Fusobacteria Antonie Van Leeuwenhoek* 1987;53:261–71. 10.1007/BF00393933 3674857

[15] Celis AI, Relman DA, Huang KC. The impact of iron and heme availability on the healthy human gut microbiome in vivo and in vitro. *Cell Chem Biol* 2023;30:110–126.e3. 10.1016/j.chembiol.2022.12.001 36603582 PMC9913275

[16] Bradshaw DJ, Marsh PD, Watson GK. et al. Role of *fusobacterium nucleatum* and coaggregation in anaerobe survival in planktonic and biofilm oral microbial communities during aeration. *Infect Immun* 1998;66:4729–32. 10.1128/IAI.66.10.4729-4732.1998 9746571 PMC108582

[17] Hoare A, Wang H, Meethil A. et al. A cross-species interaction with a symbiotic commensal enables cell-density-dependent growth and in vivo virulence of an oral pathogen. *ISME J* 2021;15:1490–504. 10.1038/s41396-020-00865-y 33372193 PMC8115154

[18] Yost S, Duran-Pinedo AE, Teles R. et al. Functional signatures of oral dysbiosis during periodontitis progression revealed by microbial metatranscriptome analysis. *Genome Med* 2015;7:27. 10.1186/s13073-015-0153-3 25918553 PMC4410737

[19] Lamster IB . Evaluation of components of gingival crevicular fluid as diagnostic tests. *Ann Periodontol* 1997;2:123–37. 10.1902/annals.1997.2.1.123 9151549

[20] Romano F, Iaderosa G, Corana M. et al. Comparing ionic profile of gingival crevicular fluid and saliva as distinctive signature of severe periodontitis. *Biomedicines.* 2022;10:687. 10.3390/biomedicines10030687 35327490 PMC8945093

[21] Cuevas-Gonzalez MV, Cuevas-Gonzalez JC, Espinosa-Cristobal LF. et al. The potential of gingival crevicular fluid as a tool for molecular diagnosis: a systematic review. *Biomed Res Int* 2024;2024:5560866. 10.1155/2024/5560866 39445210 PMC11496582

[22] Ozkavaf A, Aras H, Huri CB. et al. Relationship between the quantity of gingival crevicular fluid and clinical periodontal status. *J Oral Sci* 2000;42:231–8. 10.2334/josnusd.42.231 11269382

[23] Hatipoglu H, Yamalik N, Berberoglu A. et al. Impact of the distinct sampling area on volumetric features of gingival crevicular fluid. *J Periodontol* 2007;78:705–15. 10.1902/jop.2007.060331 17397319

[24] Lamont EI, Gadkari A, Kerns KA. et al. Modified SHI medium supports growth of a disease-state subgingival polymicrobial community in vitro. *Mol Oral Microbiol* 2021;36:37–49. 10.1111/omi.12323 33174294 PMC7984074

[25] Kinniment SL, Wimpenny JW, Adams D. et al. Development of a steady-state oral microbial biofilm community using the constant-depth film ferrnenter. *Microbiology.* 1996;142:631–8. 10.1099/13500872-142-3-631 8868438

[26] Bizhang M, Ellerbrock B, Preza D. et al. Detection of nine microorganisms from the initial carious root lesions using a TaqMan-based real-time PCR. *Oral Dis* 2011;17:642–52. 10.1111/j.1601-0825.2011.01815.x 21605286

[27] Nadkarni MA, Martin FE, Jacques NA. et al. Determination of bacterial load by real-time PCR using a broad-range (universal) probe and primers set. *Microbiology (Reading)* 2002;148:257–66. 10.1099/00221287-148-1-257 11782518

[28] Hong BY, Sobue T, Choquette L. et al. Chemotherapy-induced oral mucositis is associated with detrimental bacterial dysbiosis. *Microbiome.* 2019;7:66. 10.1186/s40168-019-0679-5 31018870 PMC6482518

[29] Schincaglia GP, Hong BY, Rosania A. et al. Clinical, immune, and microbiome traits of gingivitis and peri-implant mucositis. *J Dent Res* 2017;96:47–55. 10.1177/0022034516668847 28033066

[30] Schloss PD, Westcott SL, Ryabin T. et al. Introducing mothur: open-source, platform-independent, community-supported software for describing and comparing microbial communities. *Appl Environ Microbiol* 2009;75:7537–41. 10.1128/AEM.01541-09 19801464 PMC2786419

[31] Edgar RC, Haas BJ, Clemente JC. et al. UCHIME improves sensitivity and speed of chimera detection. *Bioinformatics.* 2011;27:2194–200. 10.1093/bioinformatics/btr381 21700674 PMC3150044

[32] Dewhirst FE, Chen T, Izard J. et al. The human oral microbiome. *J Bacteriol* 2010;192:5002–17. 10.1128/JB.00542-10 20656903 PMC2944498

[33] Wright RJ, Langille MGI. PICRUSt2-SC: an update to the reference database used for functional prediction within PICRUSt2. *Bioinformatics.* 2025;41:btaf269. 10.1093/bioinformatics/btaf269 40293718 PMC12089645

[34] Huttenhower Lab HU. https://huttenhower.sph.harvard.edu/kneaddata.

[35] Warr A, Affara N, Aken B. et al. An improved pig reference genome sequence to enable pig genetics and genomics research. *Gigascience.* 2020;9:giaa051. 10.1093/gigascience/giaa051 32543654 PMC7448572

[36] Kalbfleisch TS, Rice ES, DePriest MS. et al. Improved reference genome for the domestic horse increases assembly contiguity and composition. *Commun Biol* 2018;1:197. 10.1038/s42003-018-0199-z 30456315 PMC6240028

[37] Church DM, Schneider VA, Graves T. et al. Modernizing reference genome assemblies. *PLoS Biol* 2011;9:e1001091. 10.1371/journal.pbio.1001091 21750661 PMC3130012

[38] Langmead B, Salzberg SL. Fast gapped-read alignment with bowtie 2. *Nat Methods* 2012;9:357–9. 10.1038/nmeth.1923 22388286 PMC3322381

[39] Li H, Handsaker B, Wysoker A. et al. The sequence alignment/map format and SAMtools. *Bioinformatics.* 2009;25:2078–9. 10.1093/bioinformatics/btp352 19505943 PMC2723002

[40] Buchfink B, Reuter K, Drost HG. Sensitive protein alignments at tree-of-life scale using DIAMOND. *Nat Methods* 2021;18:366–8. 10.1038/s41592-021-01101-x 33828273 PMC8026399

[41] Ogata H, Goto S, Sato K. et al. KEGG: Kyoto encyclopedia of genes and genomes. *Nucleic Acids Res* 1999;27:29–34. 10.1093/nar/27.1.29 9847135 PMC148090

[42] Ashburner M, Ball CA, Blake JA. et al. Gene ontology: tool for the unification of biology. The gene ontology consortium. *Nat Genet* 2000;25:25–9. 10.1038/75556 10802651 PMC3037419

[43] Love MI, Huber W, Anders S. Moderated estimation of fold change and dispersion for RNA-seq data with DESeq2. *Genome Biol* 2014;15:550. 10.1186/s13059-014-0550-8 25516281 PMC4302049

[44] Supek F, Bošnjak M, Škunca N. et al. REVIGO summarizes and visualizes long lists of gene ontology terms. *PLoS One* 2011;6:e21800. 10.1371/journal.pone.0021800 21789182 PMC3138752

[45] Katajamaa M, Miettinen J, Oresic M. MZmine: toolbox for processing and visualization of mass spectrometry based molecular profile data. *Bioinformatics.* 2006;22:634–6. 10.1093/bioinformatics/btk039 16403790

[46] Wishart DS, Guo A, Oler E. et al. HMDB 5.0: the human metabolome database for 2022. *Nucleic Acids Res* 2022;50:D622–31. 10.1093/nar/gkab1062 34986597 PMC8728138

[47] Pang Z, Zhou G, Ewald J. et al. Using MetaboAnalyst 5.0 for LC-HRMS spectra processing, multi-omics integration and covariate adjustment of global metabolomics data. *Nat Protoc* 2022;17:1735–61. 10.1038/s41596-022-00710-w 35715522

[48] Simon-Soro A, Ren Z, Krom BP. et al. Polymicrobial aggregates in human saliva build the oral biofilm. *MBio.* 2022;13:e00131–22. 10.1128/mbio.00131-22 35189700 PMC8903893

[49] Gladwin MT, Shelhamer JH, Schechter AN. et al. Role of circulating nitrite and S-nitrosohemoglobin in the regulation of regional blood flow in humans. *Proc Natl Acad Sci USA* 2000;97:11482–7. 10.1073/pnas.97.21.11482 11027349 PMC17226

[50] Giovannoni G, Land JM, Keir G. et al. Adaptation of the nitrate reductase and Griess reaction methods for the measurement of serum nitrate plus nitrite levels. *Ann Clin Biochem* 1997;34:193–8. 10.1177/000456329703400212 9133256

[51] Xie H, Cai S, Lamont RJ. Environmental regulation of fimbrial gene expression in *Porphyromonas gingivalis*. *Infect Immun* 1997;65:2265–71. 10.1128/iai.65.6.2265-2271.1997 9169762 PMC175314

[52] Duran-Pinedo AE, Chen T, Teles R. et al. Community-wide transcriptome of the oral microbiome in subjects with and without periodontitis. *ISME J.* 2014;8:1659–72. 10.1038/ismej.2014.23 24599074 PMC4817619

[53] Belvin BR, Gui Q, Hutcherson JA. et al. The *Porphyromonas gingivalis* hybrid cluster protein hcp is required for growth with nitrite and survival with host cells. *Infect Immun* 2019;87:10–128. 10.1128/IAI.00572-18 PMC6434115 30670550

[54] Lewis JP, Yanamandra SS, Anaya-Bergman C. HcpR of *Porphyromonas gingivalis* is required for growth under nitrosative stress and survival within host cells. *Infect Immun* 2012;80:3319–31. 10.1128/IAI.00561-12 22778102 PMC3418757

[55] Ishiguro K, Arai T, Suzuki T. Depletion of S-adenosylmethionine impacts on ribosome biogenesis through hypomodification of a single rRNA methylation. *Nucleic Acids Res* 2019;47:4226–39. 10.1093/nar/gkz111 30799486 PMC6486555

[56] Wang SC, Frey PA. S-adenosylmethionine as an oxidant: the radical SAM superfamily. *Trends Biochem Sci* 2007;32:101–10. 10.1016/j.tibs.2007.01.002 17291766

[57] Hanzelka BL, Greenberg E. Quorum sensing in *Vibrio fischeri*: evidence that S-adenosylmethionine is the amino acid substrate for autoinducer synthesis. *J Bacteriol* 1996;178:5291–4. 10.1128/jb.178.17.5291-5294.1996 8752350 PMC178329

[58] Schauder S, Shokat K, Surette MG. et al. The LuxS family of bacterial autoinducers: biosynthesis of a novel quorum-sensing signal molecule. *Mol Microbiol* 2001;41:463–76. 10.1046/j.1365-2958.2001.02532.x 11489131

[59] Van Der Heide T, Poolman B. Osmoregulated ABC-transport system of *Lactococcus lactis* senses water stress via changes in the physical state of the membrane. *Proc Natl Acad Sci USA* 2000;97:7102–6. 10.1073/pnas.97.13.7102 10860977 PMC16506

[60] Bradshaw DJ, Marsh PD, Hodgson RJ. et al. Effects of glucose and fluoride on competition and metabolism within in vitro dental bacterial communities and biofilms. *Caries Res* 2002;36:81–6. 10.1159/000057864 12037363

[61] Weiss AS, Niedermeier LS, von Strempel A. et al. Nutritional and host environments determine community ecology and keystone species in a synthetic gut bacterial community. *Nat Commun* 2023;14:4780. 10.1038/s41467-023-40372-0 37553336 PMC10409746

[62] Goldman DA, Xue KS, Parrott AB. et al. Competition for shared resources increases dependence on initial population size during coalescence of gut microbial communities. *Proc Natl Acad Sci USA* 2025;122:e2322440122. 10.1073/pnas.2322440122 40063808 PMC11929384

[63] Bradshaw DJ, Marsh PD, Allison C. et al. Effect of oxygen, inoculum composition and flow rate on development of mixed-culture oral biofilms. *Microbiology.* 1996;142:623–9. 10.1099/13500872-142-3-623 8868437

[64] Bradshaw DJ, Homer KA, Marsh PD. et al. Metabolic cooperation in oral microbial communities during growth on mucin. *Microbiology.* 1994;140:3407–12. 10.1099/13500872-140-12-3407 7881558

[65] Huynh AH, Veith PD, McGregor NR. et al. Gingival crevicular fluid proteomes in health, gingivitis and chronic periodontitis. *J Periodontal Res* 2014;50:637–49. 10.1111/jre.12244 25439677

[66] Goodson JM . Gingival crevice fluid flow. *Periodontol* 2000;31:43–54. 10.1034/j.1600-0757.2003.03104.x 12656995

[67] Griffiths GS . Formation, collection and significance of gingival crevice fluid. *Periodontol* 2000;31:32–42. 10.1034/j.1600-0757.2003.03103.x 12656994

[68] Tollefsen T, Saltvedt E. Comparative analysis of gingival fluid and plasma by crossed immunoelectrophoresis. *J Periodontal Res* 1980;15:96–106. 10.1111/j.1600-0765.1980.tb00263.x 6445973

[69] Schenkein HA, Genco RJ. Gingival fluid and serum in periodontal diseases. I. Quantitative study of immunoglobulins, complement components, and other plasma proteins. *J Periodontol* 1977;48:772–7. 10.1902/jop.1977.48.12.772 73582

[70] Khurshid Z, Mali M, Naseem M. et al. Human gingival crevicular fluids (GCF) proteomics: an overview. *Dent J (Basel)* 2017;5:12. 10.3390/dj5010012 29563418 PMC5806989

[71] Ter Steeg PF, Van der Hoeven JS, De Jong MH. et al. Modelling the gingival pocket by enrichment of subgingival microflora in human serum in chemostats. *Microb Ecol Health Dis* 1988;1:73–84. 10.3109/08910608809140185

[72] Jorth P, Turner KH, Gumus P. et al. Metatranscriptomics of the human oral microbiome during health and disease. *MBio.* 2014;5:e01012–4. 10.1128/mBio.01012-14 24692635 PMC3977359

[73] Dixon DR, Darveau RP. Lipopolysaccharide heterogeneity: innate host responses to bacterial modification of lipid a structure. *J Dent Res* 2005;84:584–95. 10.1177/154405910508400702 15972584

[74] McDermid AS, McKee AS, Marsh PD. Effect of environmental pH on enzyme activity and growth of *Bacteroides gingivalis* W50. *Infect Immun* 1988;56:1096–100. 10.1128/iai.56.5.1096-1100.1988 3281900 PMC259768

[75] Takahashi N, Schachtele CF. Effect of pH on the growth and proteolytic activity of *Porphyromonas gingivalis* and *Bacteroides intermedius*. *J Dent Res* 1990;69:1266–9. 10.1177/00220345900690060801 2191980

[76] Zilm PS, Rogers AH. Co-adhesion and biofilm formation by *fusobacterium nucleatum* in response to growth pH. *Anaerobe.* 2007;13:146–52. 10.1016/j.anaerobe.2007.04.005 17540586

[77] Bickel M, Cimasoni G. The pH of human crevicular fluid measured by a new microanalytical technique. *J Periodontal Res* 1985;20:35–40. 10.1111/j.1600-0765.1985.tb00408.x 3156233

[78] Han YW, Wang X. Mobile microbiome: oral bacteria in extra-oral infections and inflammation. *J Dent Res* 2013;92:485–91. 10.1177/0022034513487559 23625375 PMC3654760

