# Supplementary material for: Nutritional pressure from serum amplifies dysbiotic features in an oral microbiome synthetic community
Source: ISME J. 2026 Mar 27;20(1):wrag070. doi: 10.1093/ismejo/wrag070 (PMC13140499; doi:10.1093/ismejo/wrag070)

Figure S1. Comparison of steady-state biomass in a five-species community cultured in continuous mode at two different dilution rates. Growth conditions were 5% serum, anaerobic atmosphere (5% CO_2 in N_2), 37 °C, and pH 7.15 ± 0.15. Protocol 1 (open bars): D = 0.103 h^{-1}, Td = 6.72 h, F = 51.56 mL/h. Protocol 2 (dashed bars): D = 0.0462 h^{-1}, Td = 15 h, F = 23.21 mL/h. Biomass of *Actinomyces oris*, *Streptococcus sanguinis*, *Fusobacterium nucleatum*, and *Veillonella parvula* was assessed by colony-forming units (CFU) per mL, whereas *Porphyromonas gingivalis* abundance was determined by qPCR as 16S rRNA gene copies per mL. Differences in biomass between the two protocols were evaluated using t-tests with Bonferroni multiple-testing correction.

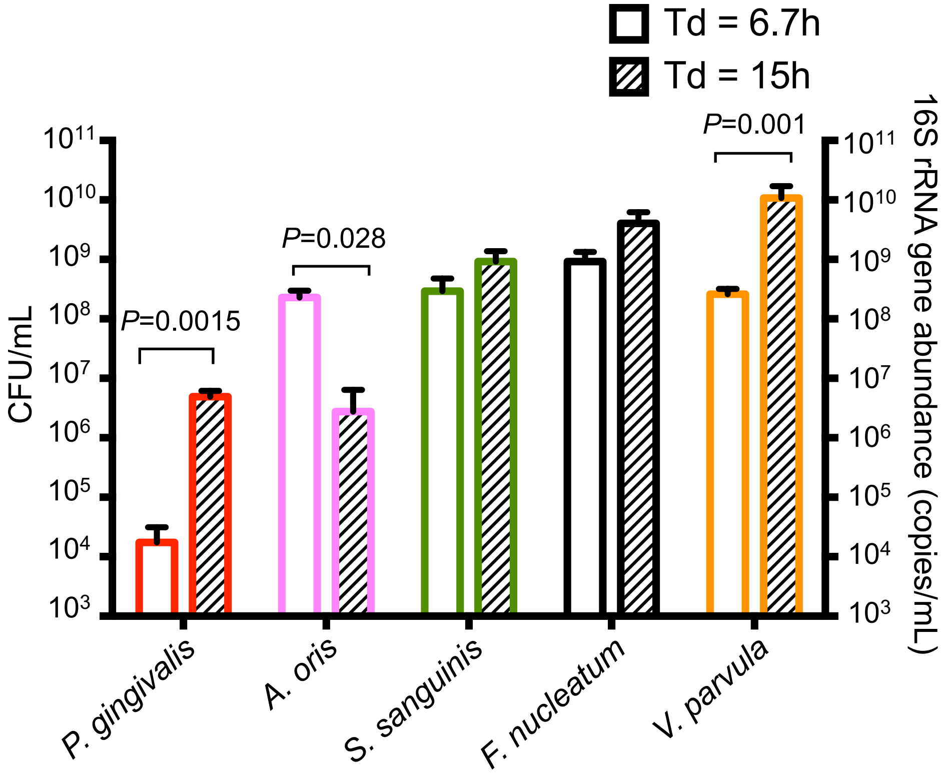

Figure S2. Reproducibility of community taxonomic composition under serum shift conditions in replicate chemostat runs. Community composition during the assembly and theoretical steady-state phases was characterized by 16S rRNA gene sequencing in two independent chemostat runs: run 1 (a) and run 2 (b). Both runs were initiated in 5% serum and subsequently shifted to 50% serum. (c) Spearman's correlations of the relative abundance of individual species between run 1 and run 2 across the full experimental period. (d) Principal coordinate analysis (PCoA) of Bray–Curtis beta-diversity of steady-state communities at different serum concentrations in run 1 and run 2. Significance of group differences was determined using PERMANOVA test with Bonferroni correction as post hoc analysis. There was no significant difference (P > 0.05) between the two runs within the same serum concentration.

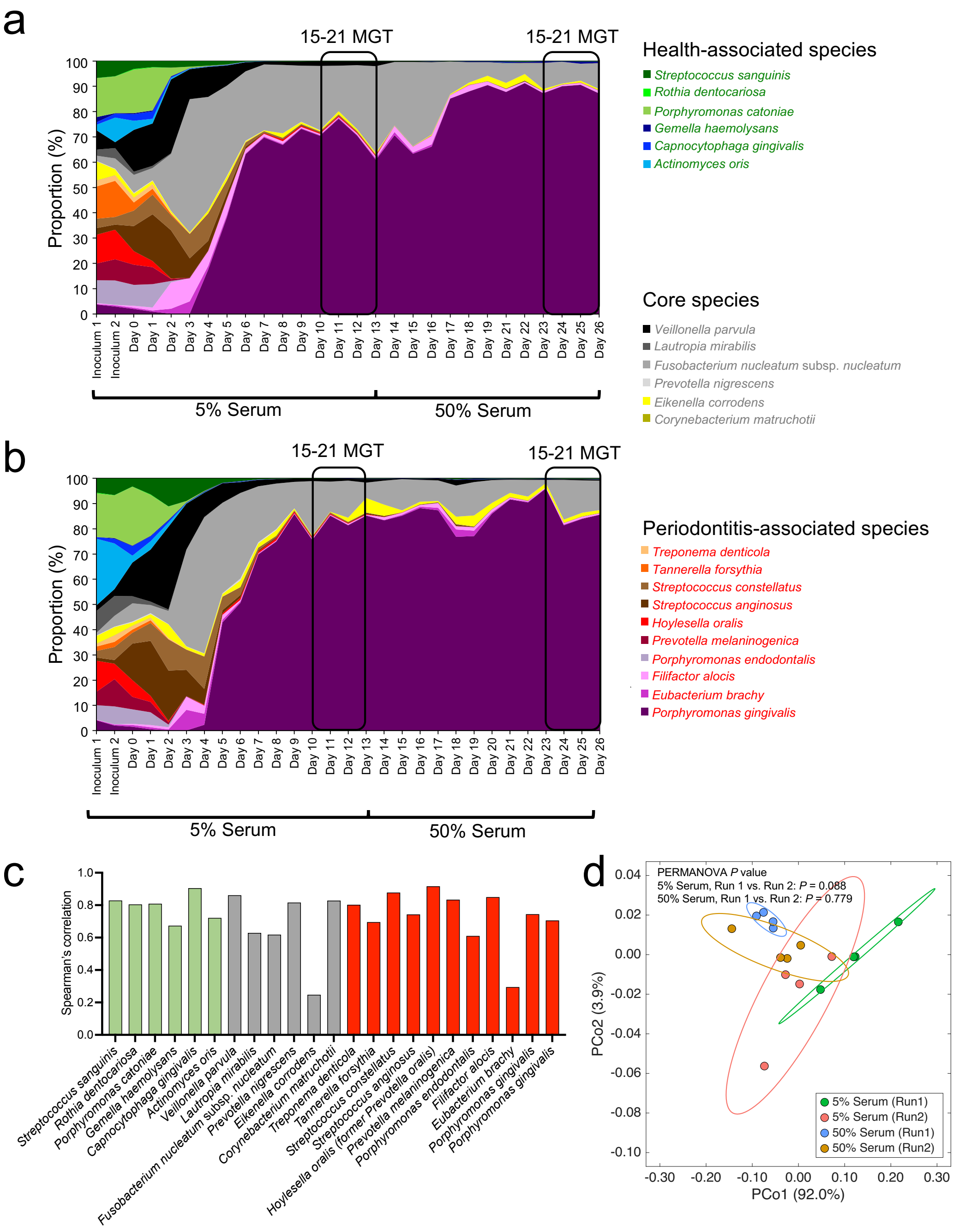

Figure S3. Fraction of genes detected for each species across different serum conditions. Bars represent the mean proportion of genes with detectable transcripts for each genome in communities grown in 0%, 5%, and 50% serum at steady state. Error bars indicate the standard deviation.

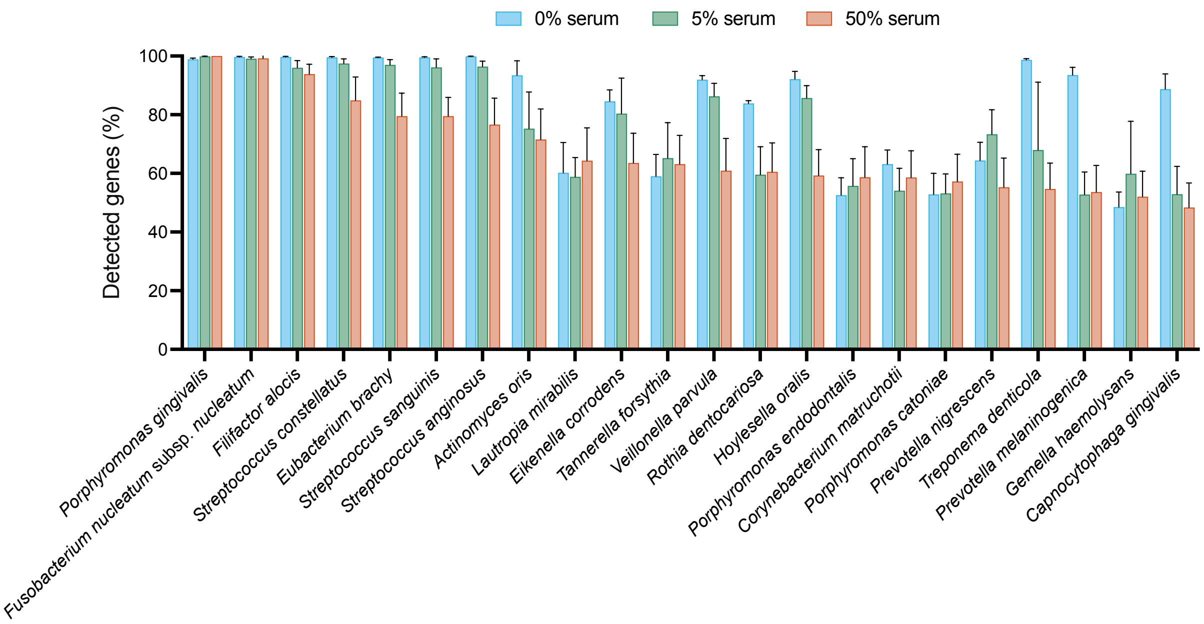

Figure S4. Effect of serum on community evenness during the assembly and acclimation period and theoretical steady state phases as characterized via 16S rRNA sequencing. (a–c) Daily evenness measured by the Shannon index over time in the three different experiments. (d) Bar plot comparing Shannon evenness at steady states across serum concentrations. Statistical significance was assessed using one-way ANOVA with Bonferroni correction for multiple comparisons (*** = $P < 0.001$).

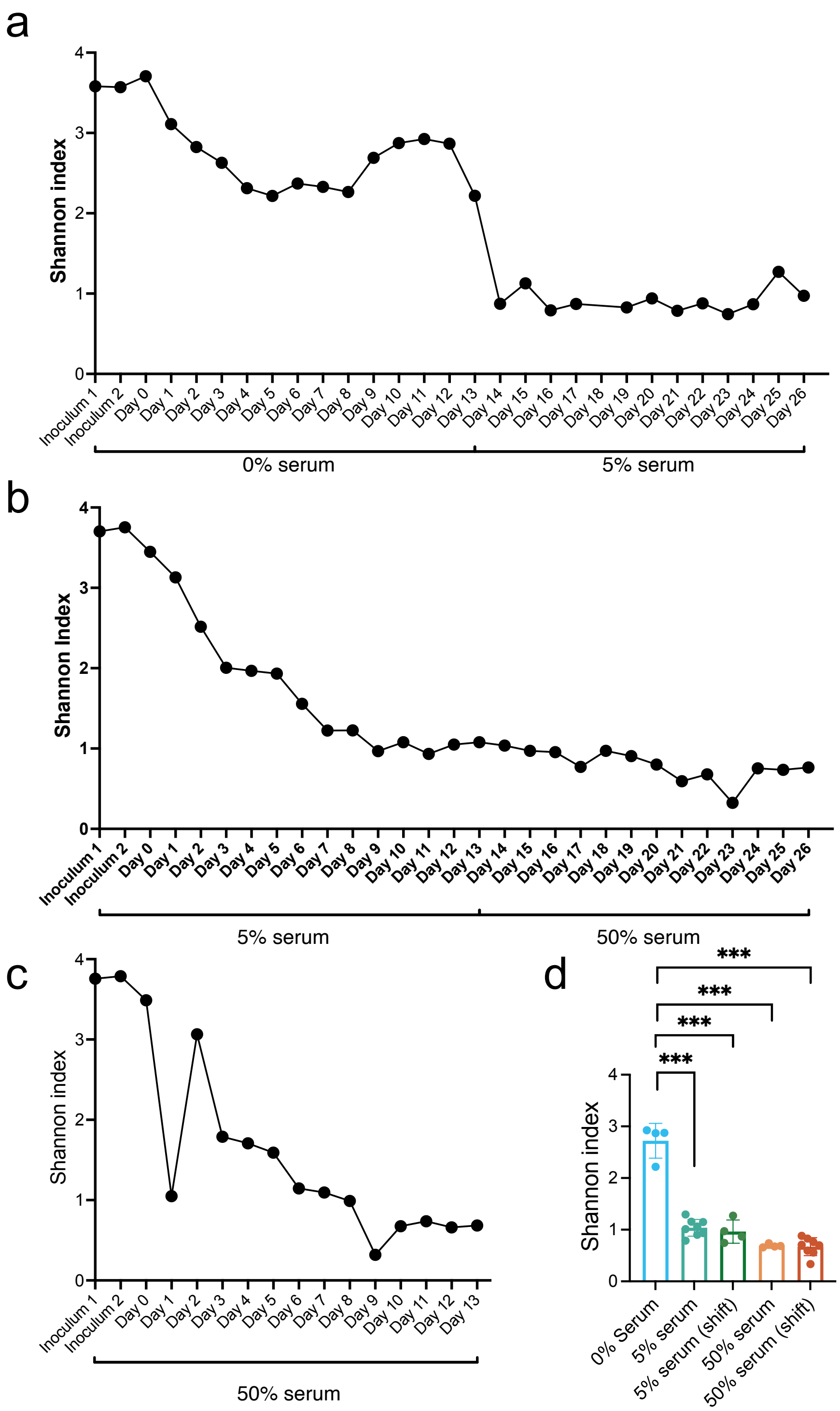

Figure S5. **Aggregate relative abundances of health-associated, core, and periodontitis-associated species at steady state across serum concentrations.** (a) Quantification based on 16S rRNA gene sequencing. (b) Quantification based on metatranscriptomic analysis. Statistical significance was determined using one-way ANOVA with Bonferroni post hoc correction (* = $P < 0.05$, ** = $P < 0.01$, *** = $P < 0.001$).

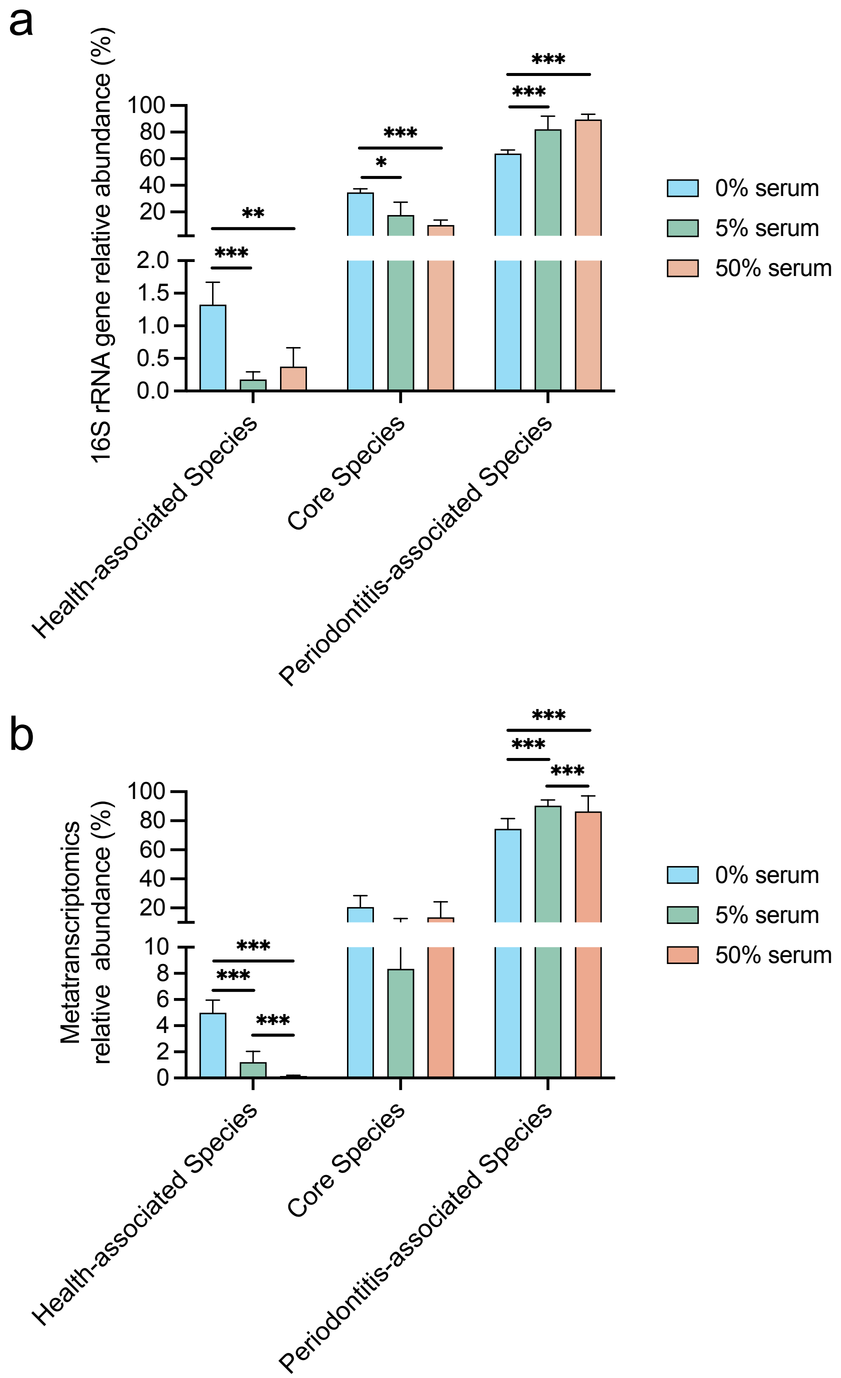

Figure S6. Heatmap plot of differentially expressed KEGG pathways across serum concentrations identified by DESeq2. Differential expression was filtered using log2FC > 0.3785 or < –0.3785 and FDR < 0.05.

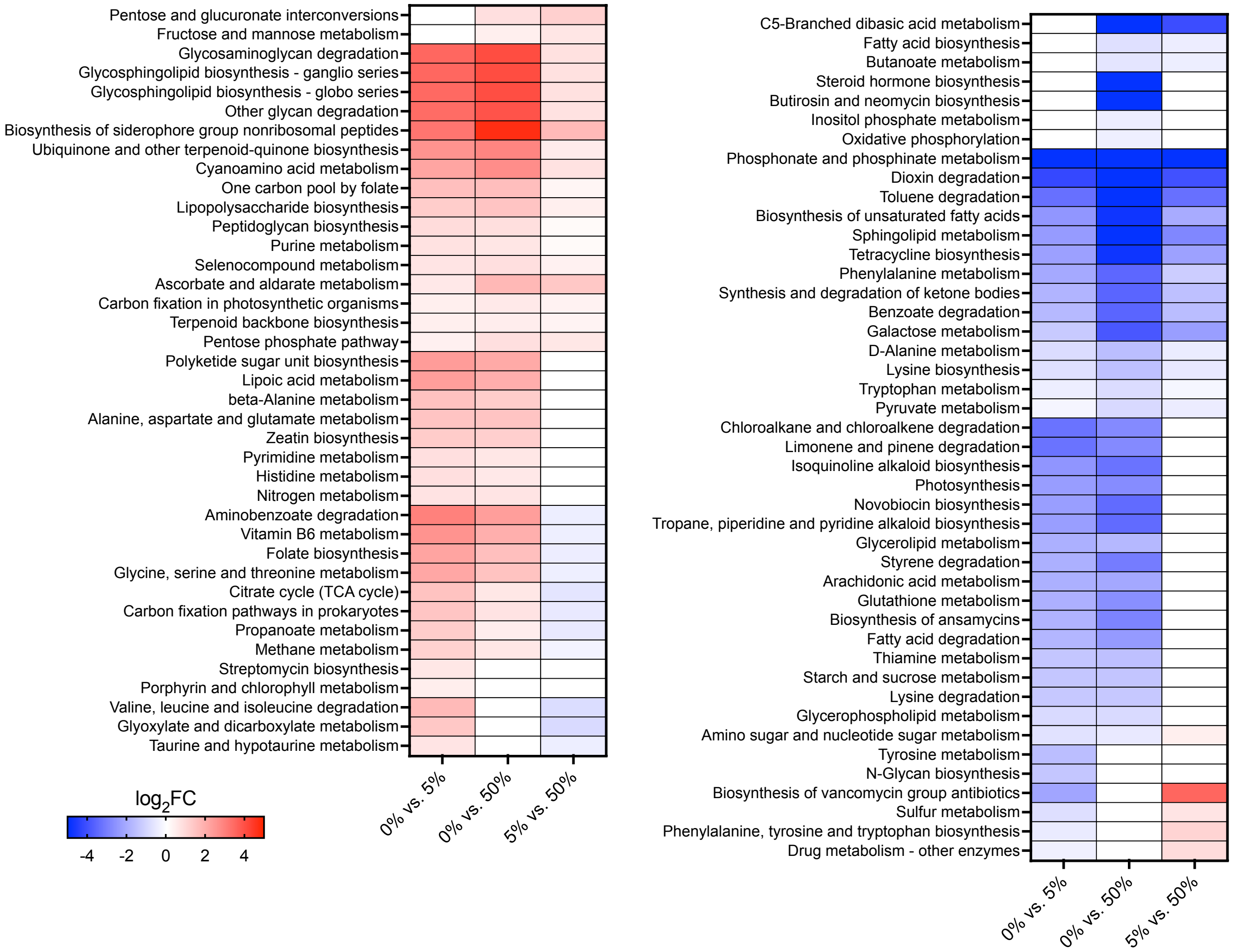

Figure S7. **Differential expression of UniRef90 gene families involved in oxidative stress across serum concentrations.** Heatmap showing log2 fold-changes (log2FC) of differentially expressed UniRef90 gene families related to the response to oxidative stress when comparing communities grown in 0%, 5%, and 50% serum. Differential expression was determined using DESeq2, with thresholds of log2FC > 0.3785 or < –0.3785 and FDR < 0.05.

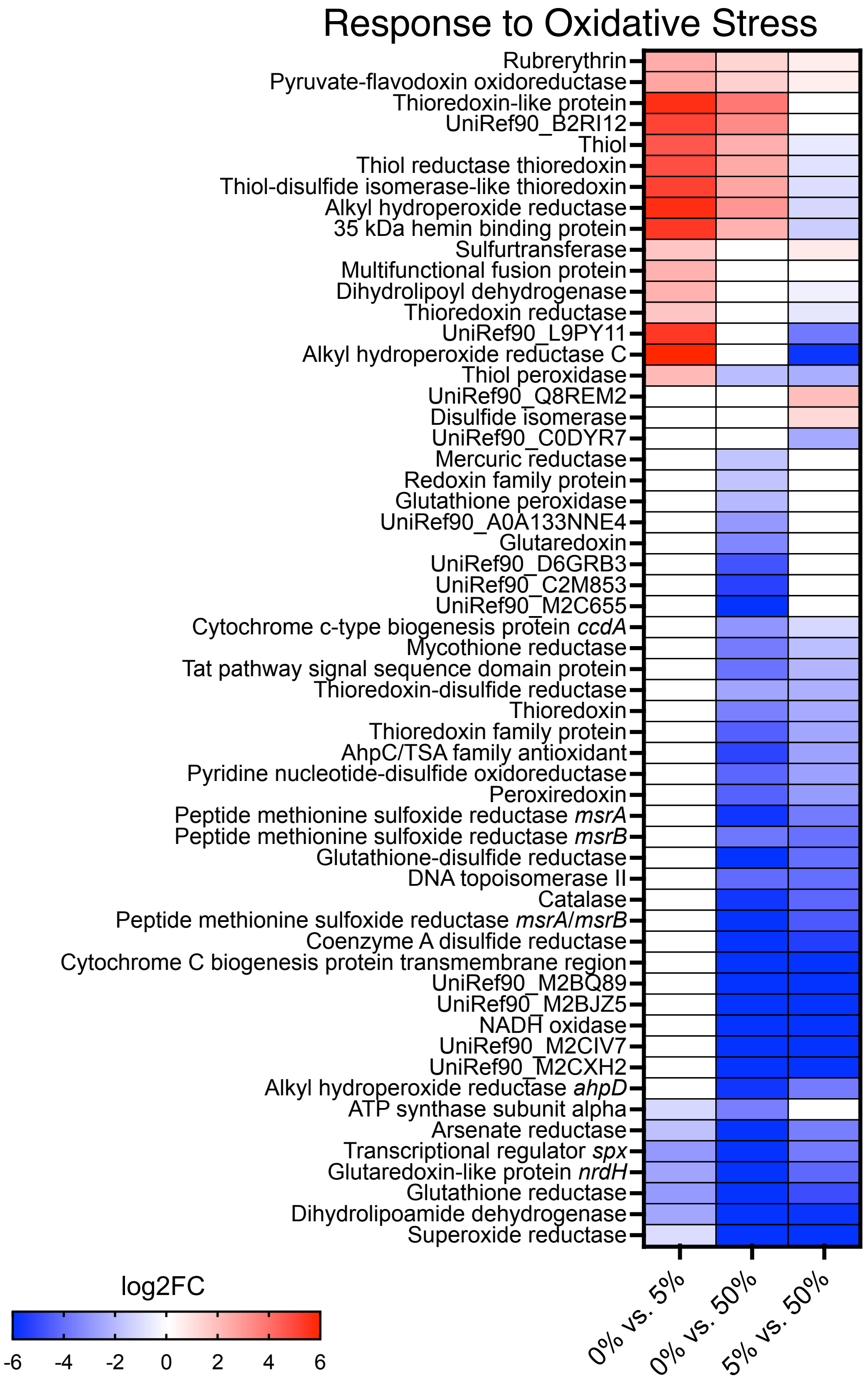

Figure S8. Oxidation-reduction potential of steady state communities in different serum concentrations. ANOVA tests were used to evaluate group-level differences with the Bonferroni method for multiple-comparison correction (*** = $P < 0.001$).

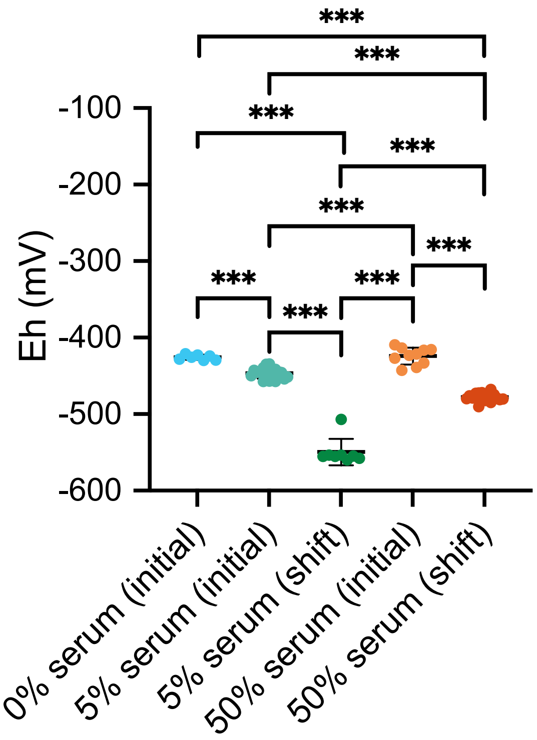

Figure S9. **Species-specific transcriptomic responses to serum.** The plots depict log2 fold-changes (log_2FC) of differentially expressed biological processes (BPs) across serum concentrations, identified by DESeq2 from six species with the greatest response to serum. Differential expression was filtered using $log_2FC > 0.3785$ or < -0.3785 and $FDR < 0.05$, with BPs unique to each species highlighted.

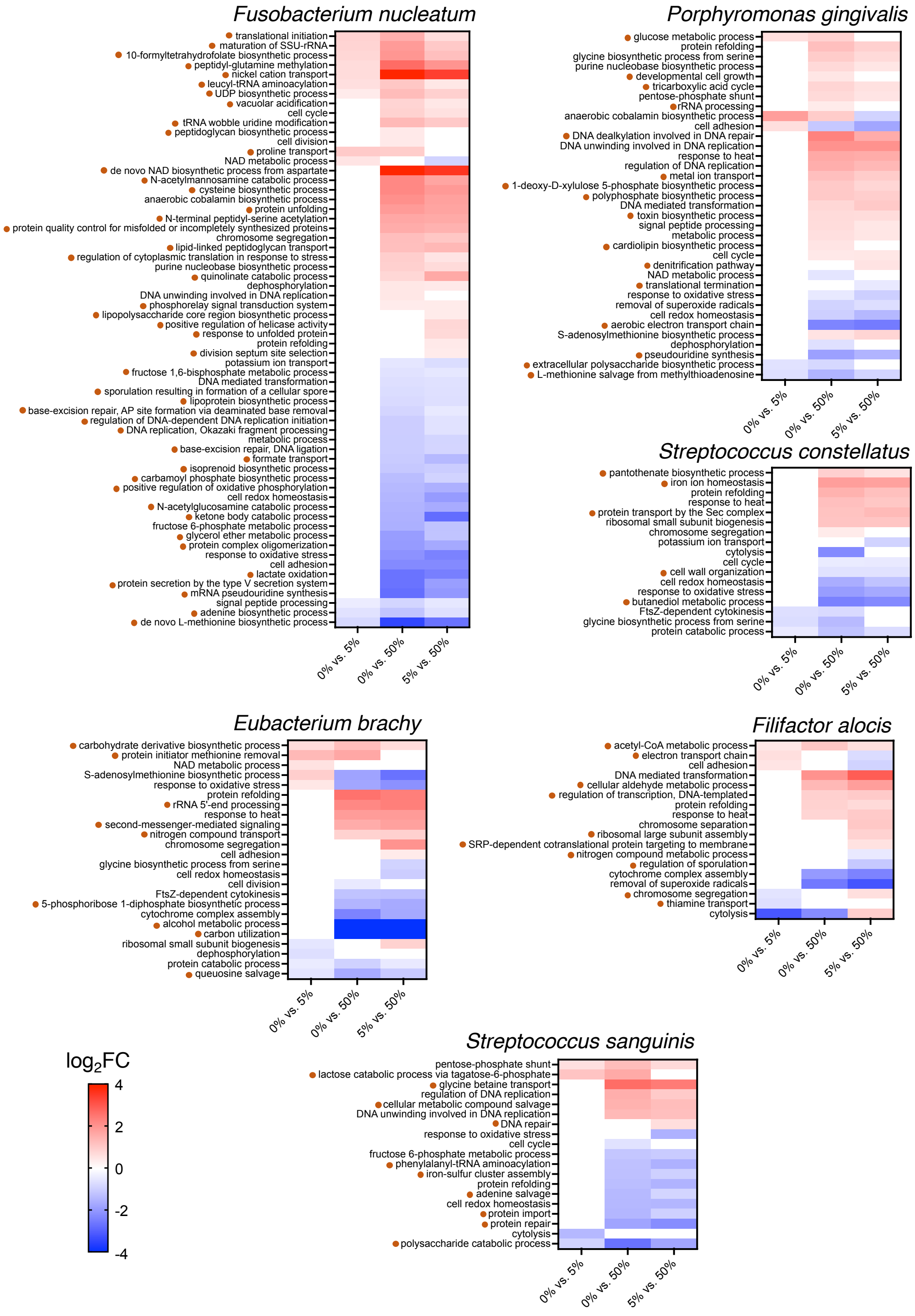

Figure S10. **Expression of nitrosative stress response genes in *Porphyromonas gingivalis* across serum concentrations.** The plots show the total number of upregulated and downregulated *P. gingivalis* genes in response to serum within the 22-spp. community, from a set of genes differentially regulated in *P. gingivalis* monocultures in response to nitrite as identified by previous *in vitro* studies (Belvin et al 2019 [53], Lewis et al 2012 [54]).

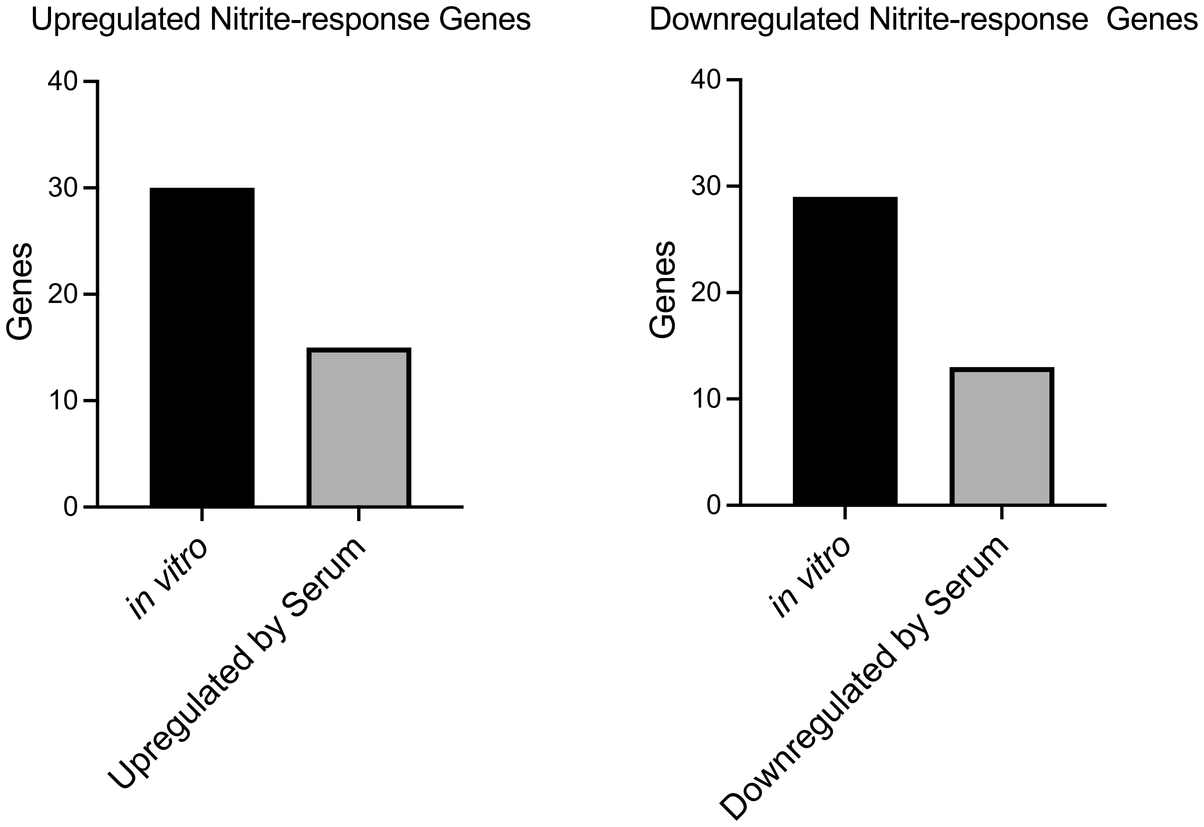

Supplement: Li_et_al_Supplementary_Figures_wrag070 [file li_et_al_supplementary_figures_wrag070.pdf]
